# Identification of promoter targets by Aureochrome 1a in the diatom *Phaeodactylum tricornutum*

**DOI:** 10.1093/jxb/erad478

**Published:** 2023-12-08

**Authors:** Soo Hyun Im, Bernard Lepetit, Niccolò Mosesso, Sandeep Shrestha, Laura Weiss, Marianne Nymark, Robert Roellig, Christian Wilhelm, Erika Isono, Peter G Kroth

**Affiliations:** Plant Ecophysiology, Department of Biology, University of Konstanz, D-78457 Konstanz, Germany; Plant Ecophysiology, Department of Biology, University of Konstanz, D-78457 Konstanz, Germany; Molecular Stress Physiology, Institute of Biological Sciences, University of Rostock, D-18059 Rostock, Germany; Plant Physiology and Biochemistry, Department of Biology, University of Konstanz, D-78457 Konstanz, Germany; Plant Ecophysiology, Department of Biology, University of Konstanz, D-78457 Konstanz, Germany; Plant Ecophysiology, Department of Biology, University of Konstanz, D-78457 Konstanz, Germany; Department of Biology, Norwegian University of Science and Technology, Trondheim, N-7491, Norway; Institute of Biology, Department of Plant Physiology, University of Leipzig, D-04103 Leipzig, Germany; Institute of Biology, Department of Plant Physiology, University of Leipzig, D-04103 Leipzig, Germany; Plant Physiology and Biochemistry, Department of Biology, University of Konstanz, D-78457 Konstanz, Germany; Plant Ecophysiology, Department of Biology, University of Konstanz, D-78457 Konstanz, Germany; Lawrence Berkeley National Laboratory, USA

**Keywords:** Aureochromes, blue light receptors, diatoms, *Phaeodactylum tricornutum*, protein–protein interactions, red-shifted antenna complex, yeast one-hybrid

## Abstract

Aureochromes (AUREOs) are unique blue light receptors and transcription factors found only in stramenopile algae. While each of the four AUREOs identified in the diatom *Phaeodactylum tricornutum* may have a specific function, PtAUREO1a has been shown to have a strong impact on overall gene regulation, when light changes from red to blue light conditions. Despite its significance, the molecular mechanism of PtAUREO1a is largely unexplored. To comprehend the overall process of gene regulation by PtAUREO1a, we conducted a series of *in vitro* and *in vivo* experiments, including pull-down assays, yeast one-hybrid experiments, and phenotypical characterization using recombinant PtAUREOs and diatom mutant lines expressing a modified *PtAureo1a* gene. We describe the distinct light absorption properties of four PtAUREOs and the formation of all combinations of their potential dimers. We demonstrate the capability of PtAUREO1a and 1b to activate the genes, *diatom-specific cyclin 2*, *PtAureo1a*, and *PtAureo1c* under both light and dark conditions. Using mutant lines expressing a modified PtAUREO1a protein with a considerably reduced light absorption, we found novel evidence that PtAUREO1a regulates the expression of PtLHCF15, which is essential for red light acclimation. Based on current knowledge, we present a working model of PtAUREO1a gene regulation properties.

## Teaser

PtAUREO1a dimerizes with other transcription factors and directly influences the expression of intermediate factors, ultimately governing overall gene regulation and cell physiology including red light acclimation.

## Introduction

Light is essential for photosynthetic organisms as a driver for photosynthesis. Therefore, these organisms must be able to respond to changes in light quality and quantity (Kianianmomeni and Hallmann, 2014). Plants and algae can sense light intensity via the redox balance of the photosynthetic electron transport in the plastid, resulting in retrograde signaling to the nucleus (Pfannschmidt, 2003). However, photoreceptors enable a more sophisticated detection of both light intensities and specific wavelengths (Galvão and Fankhauser, 2015; Jaubert *et al.*, 2017). Algae, in particular, may face rapidly changing light conditions in their environment, not only because their position in the water column depends on wave action, but also because light wavelengths are absorbed differentially by the water column and the aquatic photosynthetic organisms (Depauw *et al.*, 2012).

Diatoms are unicellular photoautotrophic algae that contribute significantly to global primary production along with carbon, nitrogen, phosphorus, and silica cycles (Armbrust, 2009). Currently, the diatom *Phaeodactylum tricornutum* is the best-studied model diatom (Bowler *et al.*, 2008; Kroth *et al.*, 2018). It has been demonstrated that blue light (BL), which is a predominant waveband in the deep water column (Depauw *et al.*, 2012), affects the physiology of *P. tricornutum*, and that specific BL receptors in this alga, aureochromes (AUREOs), are involved in regulating cell division and light acclimation (Schellenberger Costa *et al.*, 2012, 2013; Huysman *et al.*, 2013; Mann *et al.*, 2017).

AUREOs are extraordinary blue light receptors, possessing a light-, oxygen-, and voltage-sensing (LOV) domain as a sensory module that non-covalently binds an FMN chromophore. In addition, they have a basic leucine zipper (bZIP) domain as an effector domain, allowing them to directly bind to gene promoter sequences and act as transcription factors (TFs) (Jakoby *et al.*, 2002; Takahashi *et al.*, 2007; Ali *et al.*, 2016; Kroth *et al.*, 2017). In this way, AUREOs can convert light signals directly into gene regulation rather than involving a phosphorylation-based signaling cascade, as found for most other photoreceptors, such as phototropins (phots) and cryptochromes (crys) (Lin, 2002; Takahashi *et al.*, 2007; Huysman *et al.*, 2013; König *et al.*, 2017b). This bipartite structure of AUREOs, being both photoreceptors and TFs, is a desirable property for biological applications, such as optogenetics (Losi *et al.*, 2018).

In *P. tricornutum*, four AUREO genes have been identified: *PtAureo1a*, *PtAureo1b*, *PtAureo1c*, and *PtAureo2* (Depauw *et al.*, 2012; Schellenberger Costa *et al.*, 2013). While the LOV domain of class 1 PtAUREO proteins (PtAUREO1a/1b/1c) can bind the light-sensing cofactor FMN, PtAUREO2 has a mutation that inhibits FMN binding (Banerjee *et al.*, 2016a). Transcription of *PtAureo1a* and *1c* genes seem to be diurnally regulated, while *PtAureo1b* expression is light induced, and *PtAureo2* shows a basal constitutive expression without a distinct pattern (Banerjee *et al.*, 2016b). These expression patterns may indicate that the AUREOs in *P. tricornutum* perform different tasks. However, as bZIP proteins form dimers when binding to DNA (Ellenberger, 1994), potential cooperative functions of PtAUREOs by forming different dimers have been proposed. Indeed, the formation of PtAUREO1a–PtAUREO1a homodimers and PtAUREO1a–PtAUREO1c heterodimers have been verified by *in vitro* experiments (Banerjee *et al.*, 2016b).

BL induces a conformational change and dimerization of the photosensory module of PtAUREO1a, A'α-LOV-Jα (Herman *et al.*, 2013; Akiyama *et al.*, 2016), and increases PtAUREO1a–DNA binding affinity (Banerjee *et al.*, 2016b; Heintz and Schlichting, 2016). A recent transcriptomic analysis shows that PtAUREO1a plays a key role in regulating overall gene transcription (Mann *et al.*, 2020) and that PtAUREO1a impacts the cell physiology in *P. tricornutum* in response to BL illumination (Huysman *et al.*, 2013; Schellenberger Costa *et al.*, 2013; Mann *et al.*, 2017). Upon a shift from red light (RL) to BL, ~75% of genes are differentially regulated after 10 min of BL exposure in wild-type (WT) cells, while ~90% of these genes, which showed significant differences in the WT, are not differentially expressed in *PtAureo1a* gene knockout (A1aKO) lines (Mann *et al.*, 2020).

Interestingly, it was also observed that PtAUREO1a binds to DNA even in dark conditions (Banerjee *et al.*, 2016b; Heintz and Schlichting, 2016) and that PtAUREO1a may impact gene regulation in RL conditions. Several TFs are not expressed in A1aKO lines compared with the WT in RL conditions (Mann *et al.*, 2020), and *PtAureo1a* gene silencing lines exhibit different phenotypes compared with the WT in both BL and RL conditions (Schellenberger Costa *et al.*, 2013). These observations imply that gene regulation via PtAUREO1a is triggered not only by BL, but also potentially by RL or by binding other complementing factors.

So far, it remains unclear how a single protein, PtAUREO1a, which is only moderately expressed in the cell (Mann *et al.*, 2020), affects a large number of genes, leading to comprehensive gene regulation. Assuming that PtAUREO1a alone cannot regulate several thousand genes directly, it is hypothesized that PtAUREO1a may control expression of a wide range of genes in collaborative ways with other cofactors, for example by forming heterodimers (with other bZIP TFs, including other PtAUREOs). In addition, some of these directly expressed genes are suggested to encode intermediate factors, which then govern various downstream signaling cascades, resulting in broad indirect gene regulation via PtAUREO1a.

In this report, we therefore describe *in vitro* and *in vivo* experiments to comprehend the molecular mechanism of PtAUREO1a. We experimentally tested all potential dimer combinations of the four PtAUREO paralogs using a pull-down assay, and we identified genes directly regulated by PtAUREO1a and analyzed PtAUREO1a’s transcriptional activation using a modified yeast one-hybrid (Y1H) system. Lastly, to evaluate the functionality of PtAUREO1a in relation to BL perception, we generated and studied a *PtAureo1a* mutant line in which PtAUREO1a’s FMN-binding site is modified.

## Materials and methods

### Diatom cultivation and red light to blue light shift conditions

*Phaeodactylum tricornutum* (University of Texas Culture Collection, strain 646, Pt4) and other mutant lines were grown in Provasoli’s enriched F/2 seawater medium (Guillard, 1975) without added silica and a salt content of 16.5‰. Cells were cultivated in a day:night rhythm of 16 h:8 h with a light intensity of 35 µmol photons m^–2^ s^–1^ (Osram Lumilux L58W/840, Munich, Germany) at 20 °C.

For the RL to BL shift experiment, cells were cultivated in 250 ml Erlenmeyer flasks containing 150 ml of medium in continuous RL (maximum wavelength: 636 nm, 100 µmol m^–2^ s^–1^) for 12 d, followed by 1 h of BL (maximum wavelength: 440 nm, 40 µmol m^–2^ s^–1^), based on Mann *et al.* (2020) to yield the same amount of photosynthetically absorbed radiation (Qphar 30 µmol photons m^–2^ s^–1^). Fresh F/2 medium was provided every 2–3 d to avoid nutrient limitations. Cells were harvested at each time point, after 12 d of RL (t0), and 10 min (t10) and 60 min (t60) of BL exposure. About 40 ml of cells (~2–3 × 10^6^ ml^–1^) were harvested on 1.2 µm filters (Isopore; Millipore, Burlington, MA, USA). Samples were flash-frozen in liquid nitrogen and stored at –80 °C until further use.

### Expression and characterization of recombinant PtAUREO proteins

Each pET-28(+) vector, harboring individual full-length *PtAureo1a*, *PtAureo1b*, *PtAureo1c*, *PtAureo2*, and *PtBlindA1a* genes, was transformed into *Escherichia coli* Rosetta (DE3) cells (Merck Millipore, Darmstadt, Germany) for expression of the respective PtAUREO and PtBLINDA1a recombinant proteins tagged with six histidines (His6) at the N-termini. His6-fused proteins were induced and purified according to Banerjee *et al.* (2016b).

The pGEX-6P1 vectors, harboring *PtAureo1a*, *1b*, *1c*, and *2* genes, were transformed into *E. coli* Rosetta (DE3) cells to generate recombinant PtAUREO1a,1b, 1c, and 2 proteins fused with glutathione *S*-transferase (GST) at the N-terminus of the proteins. GST-fused proteins were purified in cold buffer (50 mM Tris, 100 mM NaCl, and 10% glycerol at pH 7.4) using Protino Glutathione Agarose 4B beads (Machery-Nagel, Düren, Germany).

For FMN treatment, each protein was supplemented with a 4-fold molar excess of FMN (Merck KGaA, Darmstadt, Germany) and incubated for 2 h at 4 °C in the dark. Afterwards, non-bound free FMN was removed using Zeba™ Spin Desalting Columns (7K MWCO) (Thermo Fisher Scientific, Karlsruhe, Germany). Protein buffer (50 mM Tris, 100 mM NaCl pH 7.5) was used as a reference when measuring the UV/Vis spectra of the samples without FMN treatment. The FMN-treated buffer (the same excess amount of FMN), which was also desalted on a column, was used as a reference for the measurements of the UV/Vis spectra of FMN-treated samples.

The concentration of purified His6-tagged proteins was determined using a BCA assay (Thermo Fisher Scientific, Karlsruhe, Germany). UV/Vis spectra of each PtAUREO protein equal to 30 µM were measured using an Ultrospec 8000 spectrophotometer (GE Healthcare Europe, Freiburg, Germany) from 340 nm to 500 nm with or without additional FMN treatment.

### *In vitro* protein–protein interaction assay (pull-down assay)

For *in vitro* pull-down assays, Protino Glutathione Agarose 4B beads (Macherey-Nagel, Düren, Germany) with 100 pmol of the GST fusion proteins were incubated with an equal amount (1:1 ratio) of His6 fusion proteins in buffer (50 mM Tris, 100 mM NaCl, 0.1% Triton X-100, and 10% glycerol at pH 7.4) for 1 h or 2 h at 4 °C. The beads were washed three times with cold buffer containing 0.1% Triton X-100 and three times without Triton X-100. Subsequently, proteins were eluted in 50 mM glutathione, mixed with Laemmli buffer, separated by 12.5% SDS–PAGE, and analyzed by immunoblot. His6- and GST-tagged proteins and GST protein were detected using primary and secondary antibodies listed below with the following dilution. Primary antibodies: mouse anti-Penta-His tag monoclonal antibody (1:1000) (P-21315, Thermo Fisher Scientific); rabbit anti-GST antibody (1:1,000) (Eurogentec) is a custom-made antibody as described in Vogel *et al.* (2022). Secondary antibodies: anti-mouse–horseradish peroxidase (HRP) (1:80 000) (A9044, Sigma Aldrich); anti-rabbit–HRP (1:80 000) (A0545, Sigma Aldrich). Protein–protein interactions were verified by performing three independent pull-down assays.

### Yeast strains and plasmids

The Matchmaker Gold Yeast Y1H Library Screening System (Takara Bio Europe, Saint-Germain-en-Laye, France) was used with modifications to make a TF-centered Y1H assay instead of a conventional DNA-centered assay. The Y1HGold yeast strain (*MATα*, *ura3-52*, *his3-200*, *ade2-101*, *trp1-901*, *leu2-3*,*112*, *gal4Δ*, *gal80Δ*, *met–*, *MEL1*), which was provided with the kit, was used for heterologous protein expression and interaction studies. For cloning an exogenous gene into the yeast, the expression vectors p405ADH1 (termed V1) and pGADT7 were used. For cloning the DNA of interest, the pAbAi yeast reporter vector (termed V2) was used. For this study, we deleted the minimal promoter site of V2 to insert the whole promoter site of the selected gene (~1000 bp). Detailed information about plasmids and primers used in this study are listed in Supplementary Tables S1 and S2.

### PCR and vector cloning for yeast one-hybrid assay

*Phaeodactylum tricornutum* cDNA was used as a template to amplify each isoform of the aureochrome genes using *PtAureo* gene-specific primers (Supplementary Table S2). Each amplicon was inserted into V1 using either the traditional restriction enzyme digestion/ligation or homologous recombination according to the manufacturer’s instructions (Takara Bio Europe). To heterologously express the PtAUREO1a protein fused with a yeast activation domain (AD) in yeast cells, the yeast AD sequence was cloned at the 3' end of the *PtAureo1a* cDNA. For a DNA bait in the Y1H assay, ~1000 bp upstream of the ATG codon of selected genes were amplified by PCR from *P. tricornutum* genomic DNA (gDNA) and cloned into V2. All vector constructs were verified by Sanger sequencing (Microsynth Seqlab, Göttingen, Germany) using vector-specific primers (Supplementary Table S2).

### Yeast protein extraction and western blot

Yeast protein extraction and western blots were performed to confirm the heterologous expression of PtAUREO proteins. Whole proteins were extracted from yeast, according to Zhang *et al.* (2011).

A 20 µl aliquot of each crude protein extract was separated on a 12% polyacrylamide gel (Laemmli, 1970) and blotted onto an Amersham Protran nitrocellulose membrane (GE Healthcare, Chicago, IL, USA) using a semi-dry blotting technique with Trans-Blot Turbo (Bio-Rad, Hercules, CA, USA). The expression of the PtAUREOs was detected using the respective anti-PtAUREO-specific antiserum (Agrisera AB, Vännäs, Sweden) and goat anti-rabbit IgG (H&L) with HRP-conjugated secondary antibody (Agrisera AB, Vännäs, Sweden). Roti-Lumin Plus reagent (Carl Roth, Karlsruhe, Germany) was used for chemiluminescence detection in an Odyssey Fc imaging system (Li-Cor Biosciences, Lincoln, NE, USA) with Image Studio acquisition software.

### Microscopy and image processing

For detection of PtAUREO1a localization in yeast, exponentially growing yeast cells expressing a PtAUREO1a–green fluorescent protein (GFP) fusion construct were harvested and labeled with DAPI according to the manufacturer’s protocol (Sigma-Aldrich, St. Louis, MO, USA). Images were acquired using a Zeiss LSM 700 laser scanning confocal microscope (Carl Zeiss MicroImaging GmbH, Göttingen, Germany) with a Plan-Apochromat ×63/1.4 oil DIC objective. DAPI DNA stain fluorescence and GFP fluorescence were excited at 405 nm and 488 nm, and emission was filtered with the Variable Secondary Dichroic (VSD) channel 1, which filters wavelengths shorter than 527 nm. The images were processed using ZEN (black edition) software (Carl Zeiss Microscopy, Oberkochen, Germany).

### Yeast viability test in blue light conditions

The Y1HGold yeast strain (*MATα*, *ura3-52*, *his3-200*, *ade2-101*, *trp1-901*, *leu2-3,112*, *gal4Δ*, *gal80Δ*, *met–*, *MEL1*), provided with the kit (Takara Bio Europe), was spotted on five YPDA agar plates and grown at 30 °C in dark or BL conditions. One plate was placed in dark conditions, and the other four were placed in continuous BL conditions (maximum 50 µmol photons m^–2^ s^–1^). One plate was directly exposed to the BL, and the other plates were covered with different numbers of translucent plastic foils to expose them to the same light quality but different light intensity. The growth of yeast cells was observed after 3 d of incubation.

### Yeast one-hybrid interaction assay (spot assay)

Protein–DNA interaction assays were performed based on the manufacturer’s instructions (Takara Bio Europe) and Huo *et al.* (2021). Yeast transformants were inoculated into synthetic-defined (SD)/–Leu/–Ura liquid medium, and grown for 2 d. Each day, cells were reinoculated with fresh liquid medium to avoid overgrowth. On the third day, the OD_600_ of each sample was measured with an Ultrospec 2100 pro photometer (VWR, Bruchsal, Germany). In a sterile 96-well plate, each culture was normalized to OD_600_=1 by diluting them with sterile ddH_2_O to a total volume of 300 µl. The 10-fold serial dilution of cells was made in sterile water, and 3 µl of diluted sample was spotted on SD/–Leu/–Ura selective agar plates, one with 300 ng ml^–1^ Aureobasidin A (AbA), and one without, which are further termed +AbA and –AbA, respectively. The growth of yeast cells was checked after incubating cells in the dark for 2 d at 30 °C. All interaction assays were performed independently at least twice.

### Generation of BlindA1a and Lhcf15-KO mutants

The pM9_4Compln vector harboring the WT *PtAureo1a* gene (Madhuri *et al.*, 2019) was used to generate a new vector construct with a modified *PtAureo1a* gene (Blind_V253M vector). Site-directed mutagenesis (SDM) was used to exchange GTC nucleotides to ATG using V253M_F (forward) and V253M_R (reverse) primer sets (Supplementary Table S3). This exchange eventually substitutes the amino acid valine (V) for methionine (M) within the LOV domain of PtAUREO1a (V253→M253) (Banerjee *et al.*, 2016a).

The new vector construct was sequenced for verification using BlindA1a Seq F and BlindA1a Seq R primers (Supplementary Table S3). Blind_V253M vector was complemented to the *PtAureo1a* gene knockout mutant (A1aKO9) (Serif *et al.*, 2017) via nuclear transformation using a Bio-Rad Biolistic PDS-1000/He Particle Delivery System (Bio-Rad, Hercules, CA, USA) fitted with 1350 psi rupture disks (Apt *et al.*, 1996; Zaslavskaia *et al.*, 2000; Kroth, 2007). A total of 1 × 10^8^ cells per plate were bombarded with 1.25 µg of the plasmid. Transformants were then plated on the selective agar F/2 plates containing 4 µg ml^–1^ blasticidin S (Invitrogen, Karlsruhe, Germany) and 75 µg ml^–1^ zeocin (InvivoGen, Toulouse, France).

In order to knock out the *PtLhcf15* gene, CRISPR/Cas plasmids were generated and transformed in *P. tricornutum* (strain CCMP2561) as described in Nymark *et al.* (2017). Gene mutant lines were screened using a high-resolution melting curve analysis with the *PtLhcf15* gene-specific primers (Supplementary Table S3), and further Sanger sequencing.

### Screening of BlindA1a mutants

The gDNA of each colony grown on selective plates was isolated using the Nexttec 1step DNA isolation kit (Biozym, Hessisch Oldendorf, Germany). PCR was performed with the WT *PtAureo1a* gene-specific and the pM9_4Compln vector-specific primers (Supplementary Table S3). PCR amplicons were separated on 0.8% agarose gels and isolated using the Geneclean Turbo Kit (MP Biomedicals, Eschwege, Germany) according to the manufacturer’s instructions. The purified PCR amplicon was analyzed by Sanger sequencing using sequencing primers (Microsynth Seqlab, Göttingen, Germany) (Supplementary Table S3).

Proteins were isolated from the transformants and used for western blots to detect PtBLINDA1a using anti-PtAUREO1a-specific antiserum (Agrisera AB, Vännäs, Sweden). After confirmation of gene sequence and protein expression, the transformant line was named BlindA1a mutant.

### Quantitative PCR

Quantitative PCR (qPCR) was performed to compare gene expression changes upon RL to BL shift conditions. Primers used for qPCR are listed in Supplementary Table S4. qPCR was conducted with a QuantStudio 3 Real-Time PCR system (Applied Biosystems, California, USA) using a two-step protocol as described in Mann *et al.* (2020). Cycle threshold values and gene amplification efficiencies were calculated from PCR Miner 4.0 (Zhao and Fernald, 2005). The Phatr3_J47943.t1 gene was used as a reference gene as it is stably expressed under RL to BL shift conditions (Mann *et al.*, 2020). Relative transcript levels of each gene were normalized using a reference gene according to Pfaffl (2001) and Pfaffl *et al.* (2002). The ΔΔCt analysis was used to calculate gene expression fold changes between BL and RL conditions (gene expression in BL/gene expression in RL) (Livak and Schmittgen, 2001). The relative expression software tool (REST) was used to determine statistical differences in gene expression among samples (Pfaffl *et al.*, 2002). This tool performs statistical analysis for the relative expression in real-time PCR using a pair-wise fixed reallocation randomization test (Pfaffl *et al.*, 2002).

### Non-photochemical quenching analyses

A 6 ml aliquot of cultures in the mid-exponential phase were harvested by centrifugation at 5000 *g* for 5 min. Then 100 µl of methanol were added to the pellet and vortexed for 20 s followed by adding 900 µl of acetone and vortexing for 10 s. After centrifugation for 2 min at 16 000 *g* in a microcentrifuge 5424 (Eppendorf, Wesseling-Berzdorf, Germany), Chl *a* absorbance was determined spectrophotometrically using the Jeffrey and Humphrey formula (Jeffrey and Humphrey, 1975). Cultures were then adjusted to a *Chl a* content of 2 μg ml^–1^, and non-photochemical quenching (NPQ) was measured using PAM Imaging (Heinz Walz GmbH, Effeltrich, Germany). NPQ was induced for ~6 min at 300 μmol photons m^–2^ s^–1^ blue actinic light, and a saturating pulse with maximum intensity (~3000 μmol photons m^–2^ s^–1^, width 800 ms) was applied every 20 s to determine fluorescence with closed photosystems (*F*_m_, *F*_m_'). The calculation of NPQ was done as *F*_m_/*F*_m_'–1 using the fluorescence value at the first saturating light flash in the light as initial *F*_m_.

### Fluorescence emission spectra

The WT, A1aKO9, BlindK32, and BlindK49 were cultivated in RL conditions for 12 d with regular inoculation with the fresh F/2 medium. On the 12th day, each sample was adjusted to a Chl *a* content of 2 μg ml^–1^. Room temperature fluorescence emission spectra were measured using FluoroMax-4 (HORIBA, Oberursel, Germany) in the spectral range of 600–800 nm (slit width 2 nm) with an excitation wavelength of 435 nm (slit width 3 nm), and including internal correction factors. In the case of WT and Lhcf15-KO lines, the room temperature fluorescence emission spectra of cells cultivated under white light conditions (35 mol photons m^–2^ s^–1^) and later under RL conditions for 9 d were compared between WT and Lhcf15-KO lines.

## Results

### PtAUREO paralogs interact with each other

As dimerization of bZIP TFs is crucial for gene regulation (Ellenberger, 1994; Schütze *et al.*, 2008), we investigated the potential dimerization of the four PtAUREO protein paralogs (PtAUREO1a, 1b, 1c, and 2) using pull-down assays. We used full-length recombinant PtAUREO proteins fused with GST and His6 tags. GST-tagged PtAUREOs (GST–PtAUREOs) were used as bait, and His6-tagged PtAUREOs (His6-PtAUREOs) as prey. The GST protein (28 kDa) served as negative control. The recombinant proteins are expressed correctly, as verified with Coomassie blue staining (Supplementary Fig. S1; Supplementary Table S5). Truncated protein products can be found in GST-tagged proteins (e.g. GST–PtAUREO2), which may occur when expressing large proteins fused to GST at the N-terminus.

**Fig. 1.**
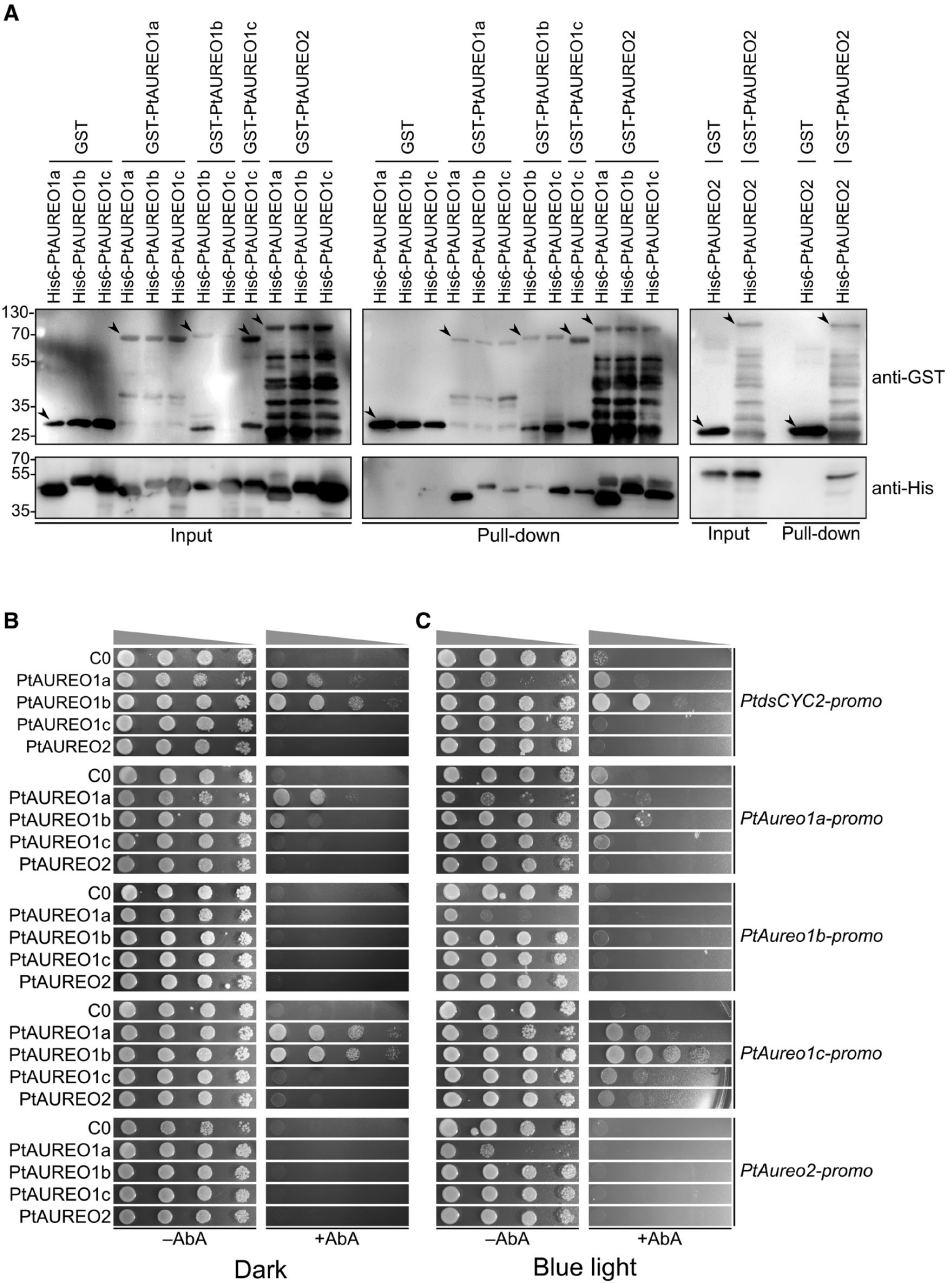
Protein–protein and protein–DNA interactions of four PtAUREOs and the promoter sites of *PtAureo* genes (*gene name-promo*). (A) Pull-down assays of PtAUREOs. The upper blot and the lower blot are detected using the anti-GST antibody and anti-His tag antibody, respectively. The respective PtAUREOs tagged with GST (GST–PtAUREO) and 6×histidine (His6-PtAUREO) were incubated and are shown in the Input blot. The GST protein alone and the respective His6-PtAUREO combination are negative controls. The arrows indicate GST alone or respective GST–PtAUREO proteins. After protein incubation, GST–PtAUREOs and the bound His6-PtAUREOs were pulled down using 50 mM glutathione. Protein–protein interactions were verified by performing three independent pull-down assays, while one representative result is shown. (B and C) The protein–DNA interaction (PDI) assay in the dark (B), and under continuous blue light conditions (C). The names of the proteins expressed in each yeast strain are listed on the left, and the gene promoter names are listed on the right. C0 is a negative control, a yeast strain with the respective promoter site without any PtAUREO protein expression. Yeast strains were spotted in serial dilution on SD/–Leu/–Ura plates with or without AbA antibiotic (+AbA or –AbA). Cell growth on the –AbA plate indicates a healthy state of yeast cells with the respective protein and promoter site. Cell growth on the +AbA plate indicates the interaction between the respective PtAUREO and the promoter site. Every interaction assay was compared with the negative control to verify the protein–DNA interaction. The interaction between PtAUREO1a and *PtdsCYC2-promo* is a positive control.

Bait and prey proteins were incubated and detected using anti-GST and anti-His tag antibodies, respectively (Fig. 1A, left panel). Detection of all bait proteins (GST–PtAUREOs or GST protein alone) on the pull-down blot indicates a successful elution of the GST protein (Fig. 1A, pull-down blot of the middle and right panel).

No His6-PtAUREOs were detected in the pull-down blot when incubated with the GST protein only, whereas we could experimentally verify the formation of all 10 possible PtAUREO homo-/heterodimers *in vitro*, as all the His6-PtAUREOs were detected from the pull-down blot, which had been incubated with the respective GST–PtAUREOs (Fig. 1A, pull-down blot of the middle and right panel). Even though all proteins were incubated with an equal molar ratio, the intensities of the prey protein bands (His6-PtAUREO) on the blot vary depending on the bait protein partner (GST–PtAUREO). For example, we observed that band intensities of eluted His6-PtAUREO1a, 1b, and 1c prey proteins vary when incubated with GST–PtAUREO1a bait protein. Notably, the eluted His6–PtAUREO1b and 1c proteins exhibited weaker band intensities compared with His6-PtAUREO1a (Fig. 1A, middle panel). Similarly, when His6-PtAUREO1b and 1c were incubated with GST–PtAUREO1b, respectively, eluted His6-PtAUREO1c shows a stronger band intensity than His6-PtAUREO1b (Fig. 1A, middle panel). On the other hand, when His6-PtAUREO1a, 1b, 1c, and 2 were incubated with GST–PtAUREO2, the eluted prey protein band intensities are comparable with each other and even comparable with those of the input proteins (Fig. 1A).

### Studying the interaction of PtAUREO proteins with individual promoter sites using a modified yeast one-hybrid system

After confirming the interactions among PtAUREO proteins, we investigated the potential binding of individual PtAUREOs to different promoter sites. We generated yeast strains that individually express either PtAUREO1a, 1b, 1c, or 2, and verified the correct expression of the proteins by western blot (Supplementary Fig. S2). In the next step, we introduced vectors containing gene promoters (a range of ~1000 bp upstream of each *PtAureo* gene) to each PtAUREO-expressing yeast strain (termed A1a, A1b, A1c, and A2 yeast) and plated the respective strains on AbA-containing antibiotic plates. In the case of successful interaction of PtAUREO proteins and a promoter, the expression of the AbA resistance gene should be initiated, resulting in colony formation on plates containing AbA (+AbA). To test the system, we performed an interaction assay using a yeast strain expressing PtAUREO1a and a promoter site of the *PtdsCYC2* gene (*PtdsCYC2-promo*), for which a successful interaction of PtAUREO1a and the *PtdsCYC2-promo* had already been shown (Huysman *et al.*, 2013). Strong colony growth of A1a yeast with *PtdsCYC2-promo* was observed on +AbA plates, while no growth was detected for the negative control (C0 yeast with *PtdsCYC2-promo*) (Fig. 1B). We then tested the interaction of other PtAUREOs with *PtdsCYC2-promo* and observed an even stronger growth response of A1b yeast (Fig. 1B), while no growth was observed for A1c and A2 yeast. When testing the potential interactions of the four PtAUREO proteins with their corresponding *PtAureo* gene promoter regions, we found that both PtAUREO1a and PtAUREO1b interact with the promoter sites of *PtAureo1a* and *PtAureo1c* genes (Fig. 1B). PtAUREO1c and PtAUREO2 showed no interaction with any of the four promoter regions.

Since aureochromes are BL receptors known to change protein conformation upon BL exposure (Herman *et al.*, 2013; Banerjee *et al.*, 2016b), we tested whether BL conditions may increase the interaction, which would be indicated by enhanced growth of the yeast cells. Before the interaction assay, we assessed the viability of the yeast cells in continuous BL conditions. The WT yeast cells were spotted on five different YPDA (yeast extract–peptone–dextrose–adenine) agar medium plates and grown under different intensities of BL (0–50 µmol m^–2^ s^–1^) (Supplementary Fig. S3). No noticeable differences in growth between yeast cells grown in the dark and BL conditions were observed (Supplementary Fig. S3). Then, we carried out PtAUREO/*PtAureo-promo* interaction assays under continuous BL conditions (50 µmol m^–2^ s^–1^). We observed identical responses compared with the dark incubation (Fig. 1B, C), except for a slight growth of A1c and A2 yeast with *PtAureo1c-promo*, which however is not indicative enough for a protein–DNA interaction (Fig. 1C). Therefore, we performed subsequent experiments in dark/dim conditions.

**Fig. 2.**
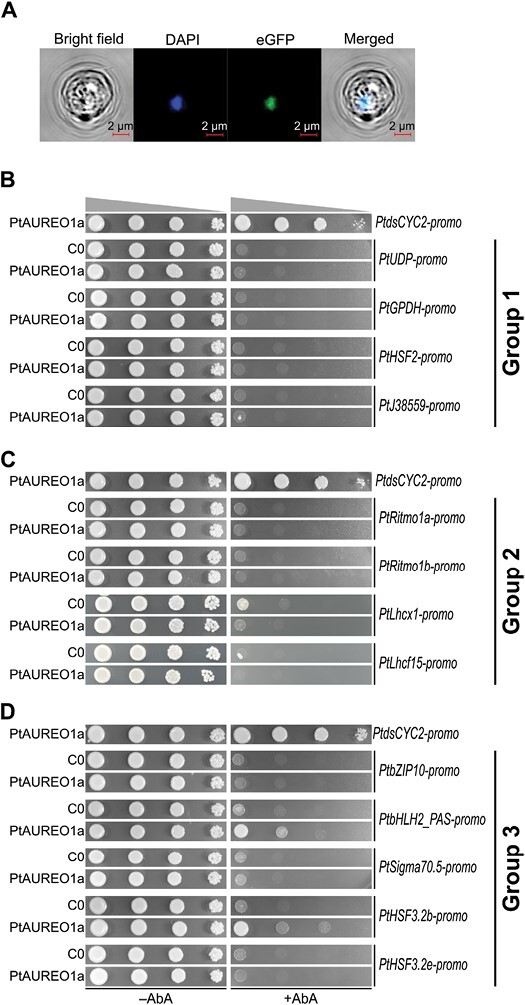
Interaction assay of the PtAUREO1a protein with the promoter sites of genes (*gene name-promo*) that are highly affected by PtAUREO1a. (A) Localization of GFP-fused PtAUREO1a in the A1a yeast strain. From left to right: bright field, nucleus staining with DAPI (blue), eGFP fluorescence (green), and a merge of all channels. Scale bar is 2 µm. (B–D) Y1H assay result. The name of the protein expressed in the yeast strain is listed on the left, and the gene promoter names are listed on the right. Yeast strains were spotted in serial dilution on SD/–Leu/–Ura plates with or without AbA antibiotic (+AbA or –AbA). C0 is a negative control, a yeast strain with the respective promoter site without any PtAUREO protein expression. A yeast strain with PtAUREO1a and *PtdsCYC2-promo* is a positive control. Growth on the +AbA plate indicates positive interaction between protein and DNA. The promoter sites of (B) four genes that are highly affected by PtAUREO1a; (C) genes that are responsible for a specific physiological feature; (D) selected transcription factor genes.

We further attempted to identify other genes that may be directly regulated by PtAUREO1a. First, we confirmed that PtAUREO1a is expressed and located in the yeast nucleus by tagging GFP to heterologously expressed PtAUREO1a (Fig. 2A). We selected three groups of genes potentially regulated by PtAUREO1a (Fig. 2), based on transcriptomic data (Mann *et al.*, 2020; Supplementary Table S6): group 1 includes UDP-glucose-pyrophosphorylase (*PtUDP*), glycerol-3-phosphate dehydrogenase (*PtGPDH*), heat shock factor 2 (*PtHSF2*), and a gene of unknown function Phatr3_J38559 (*PtJ38559*). These four genes exhibit strong changes of expression in response to a RL to BL shift only in the WT but not in *PtAureo1a* knockout mutants (Mann *et al.*, 2020). Group 2 genes play critical roles in expressing physiological phenotypes in the WT in the presence of PtAUREO1a: previous work indicated that A1aKO9 displays pronounced differences in NPQ induction (a photoprotection mechanism) and biological rhythms compared with the WT (Serif *et al.*, 2017; Madhuri, 2020). Therefore, we selected the promoter sites of *PtLhcx1*, which is responsible for NPQ induction (Bailleul *et al.*, 2010; Buck *et al.*, 2019), and of *PtRitmo1a* and *PtRitmo1b*, which play a critical role in regulating diel biological rhythms (Annunziata *et al.*, 2019). Moreover, to investigate any influence of PtAUREO1a in RL conditions, we included the promoter site of *PtLhcf15,* which encodes a key antenna complex protein that is critical for RL acclimation (Herbstová *et al.*, 2015). Group 3 includes genes of TFs that potentially transmit a BL response of AUREOs to downstream elements. We selected five TF genes showing ~4-fold increases in the WT after 10 min of BL exposure but much lower expression changes in A1aKO9 (Mann *et al.*, 2020) (Supplementary Table S6).

The interaction assays indicate that none of the promotor sites of group 1 (*PtUDP-promo*, *PtGPDH-promo*, *PtHSF2-promo*, and *PtJ38559-promo*) (Fig. 2B) and group 2 genes (*PtRitmo1a-promo*, *PtRitmo1b-promo*, *PtLhcx1-promo*, and *PtLhcf15-promo*) (Fig. 2C) interacts with PtAUREO1a directly. However, we found that the promoter sites of two TF genes, *PtbHLH2_PAS-promo* and *PtHSF3.2b-promo*, interact with PtAUREO1a (Fig. 2D).

Typically, Y1H assays focus on the interaction between the protein and DNA. Therefore, when protein is heterologously expressed in yeast, it is fused to a yeast AD, which is required to initiate the transcription of the AbA resistance gene. As AUREOs themselves are TFs possessing their own AD, we originally eliminated the yeast AD, ensuring that the native domain structure of AUREO is not disturbed. Although we observed that native states of PtAUREO1a can initiate gene transcription in yeast, we tested whether an additional yeast AD may alter the results. Therefore, we additionally generated a yeast strain that expresses PtAUREO1a fused to a yeast AD at the C-terminus (A1aAD yeast).

The corresponding interaction experiments with A1aAD yeast revealed only minor differences compared with A1a yeast (Supplementary Fig. S4). The similar growth of A1a and A1aAD yeast with *PtdsCYC2-promo* on an +AbA plate indicates that PtAUREO1a and PtAUREO1a-AD interacted with the *PtdsCYC2-promo* to a similar degree (Supplementary Fig. S4A). Surprisingly, A1aAD yeast in combination with each of *PtAureo1a-promo* and *PtAureo1c-promo* did not grow on +AbA, while these two promoters showed a strong interaction with PtAUREO1a alone (cf. Fig. 1B), indicating that the yeast AD may hinder AUREO activity (Supplementary Fig. S4A). PtAUREO1a-AD also did not interact with any of the promoter sites of groups 1 and 2 (Supplementary Fig. S4B, C), which is consistent with PtAUREO1a. However, with respect to TF promoters (group 3), an interaction between PtAUREO1a-AD and *PtbZIP10-promo* was observed (Supplementary Fig. S4D), which was not found with PtAUREO1a (cf. Fig. 2D). Furthermore, stronger growth of A1aAD yeast with *PtHSF3.2b-promo* was observed compared with the A1a yeast with *PtHSF3.2b-promo* (Supplementary Fig. S4D).

### Assessment of the light perception module of PtAUREO1a by analyzing BlindA1a mutants

**Fig. 3.**
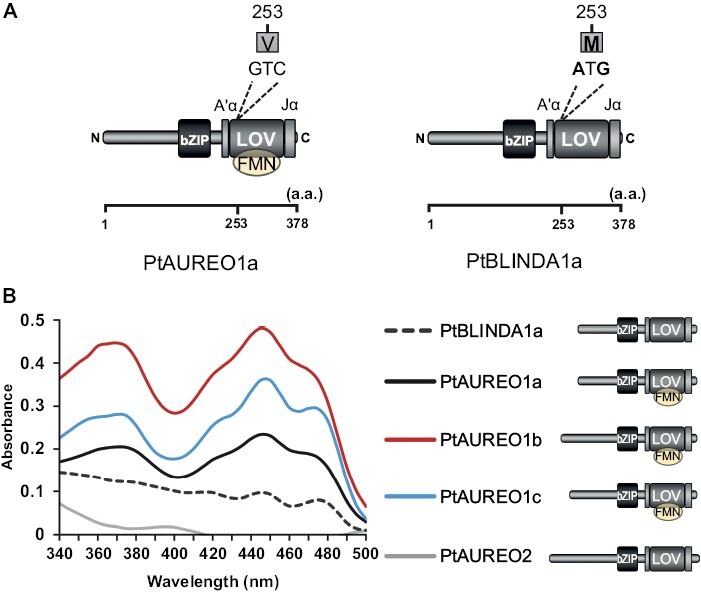
Distinct light absorption properties of each PtAUREO. (A) Scheme of full-length PtAUREO1a and PtBLINDA1a. Valine is substituted by methionine (V253M) in the LOV domain of PtBLINDA1a. (B) UV/Vis spectra of full-length recombinant PtAUREOs and PtBLINDA1a. All proteins were normalized to the same molarity (30 µM). A schematic depiction of full-length PtAUREOs and PtBLINDA1a is shown on the right. The scheme of aureochrome is reprinted (adapted and modifed) with permission from Herman *et al.*(2013). Blue-light-induced unfolding of the Jα helix allows for the dimerization of aureochrome-LOV from the diatom *Phaeodactylum tricornutum*. Biochemistry 52, 3094–3101. Copyright 2013 American Chemical Society.

As we had observed that PtAUREO1a initiates gene transcription in the dark in a heterologous system, we further investigated the relevance of the light perception of PtAUREO1a for its functionality *in vivo* in *P. tricornutum*. As a mutation in the LOV domain of PtAUREO1a (V253M) hinders FMN binding (Banerjee *et al.* 2016a) (Fig. 3A), we tested such modified proteins (PtBLINDA1a) *in vitro* and *in vivo*. We analyzed its absorption properties and compared it with all full-length recombinant PtAUREOs expressed in *E. coli*. To compensate for potential loss of the non-covalently bound chromophore during the protein purification procedure or eventually reduced FMN availability in *E. coli*, we reconstituted FMN loads by adding free FMN in excess after protein purification, followed by subsequent removal of unbound FMN. UV/Vis spectroscopy of PtAUREO1a, 1b, and 1c proteins reveals maximum absorption peaks at 447–448 nm, which reflects the typical absorption spectra of a flavoprotein and indicates the absorption maximum of the ground state of FMN in the LOV domain (445–450 nm) (Zikihara *et al.*, 2006), while no such peak is found for PtAUREO2 (Fig. 3B). Furthermore, PtAUREO1b and PtAUREO1c both show double peaks near the UV/A region of the spectrum at ~350–370 nm, which is typical for the phot-LOV1-like spectrum, while PtAUREO1a has only one peak at ~375 nm, which is a typical phot-LOV2-like spectrum (Raffelberg *et al.*, 2013).

PtAUREO1b showed the highest light absorption, followed by PtAUREO1c and PtAUREO1a, indicating different FMN occupancies of PtAUREOs as well as the potential differences in FMN binding strengths. Although PtBLINDA1a displays maximum absorption peaks at 418 nm and 446 nm, indicating that a certain share of this protein does bind to FMN, it has a strongly reduced absorption compared with PtAUREO1a, 1b, and 1c (Fig. 3).

**Fig. 4.**
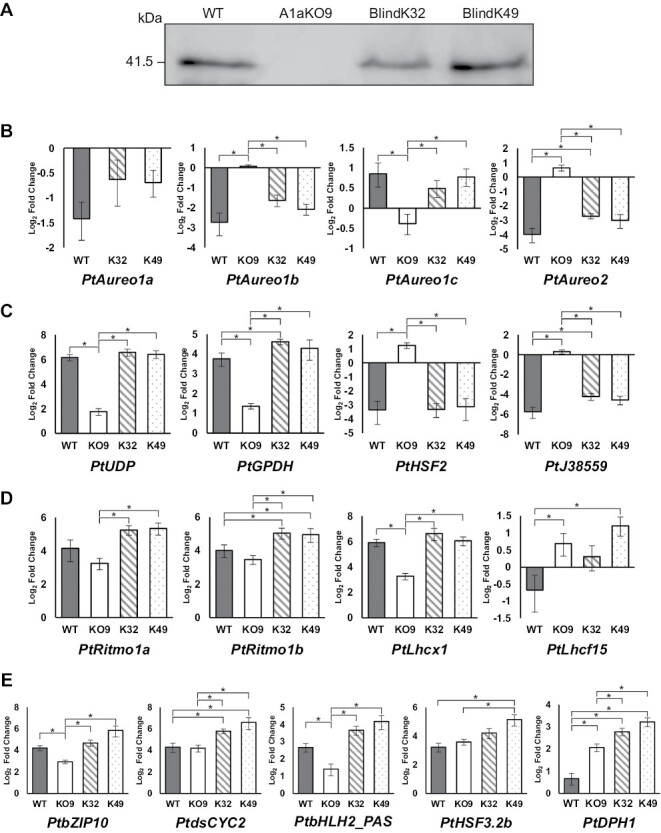
Effect of the PtBLINDA1a protein on gene expression. (A) Western blot of PtAUREO1a protein in the WT and of PtBLINDA1a protein in two BlindA1a mutants using the anti-PtAUREO1a-specific antiserum. (B–E) Gene expression changes of selected genes after a shift from 12 d of red light incubation to 10 min of short blue light exposure. Mean values of WT (gray bar), A1aKO9 (KO9, white open bar), BlindK32 (K32, bar with diagonal stripes), and BlindK49 (K49, dotted bar) are shown with the SE (*n*=4 for WT, KO9, K32, and *n*=3 for K49). Statistically significant differences between samples were determined using a pair-wise fixed reallocation randomization test and marked with an asterisk (*P≤0.05). (B) *PtAureo* genes. (C) Genes that are highly affected by PtAUREO1a. (D) Genes that are responsible for a specific physiological feature. (E) Four genes that are directly regulated by PtAUREO1a and the phytochrome gene (*PtDPH1*).

We successfully generated independent BlindA1a mutants in the A1aKO9 knockout line (Fig. 4; Supplementary Fig. S5), of which two strains (BlindK32 and BlindK49) were chosen for further experiments as the expression of PtBLINDA1a protein in these lines is as strong as that of PtAUREO1a in the WT line (Fig. 4A). In order to assess the correlation between PtAUREO1a’s light sensing and its functionality, we performed an RL to BL shift experiment under identical conditions as described in Mann *et al.* (2020). These conditions were originally chosen to achieve the maximum impact of BL when comparing WT and *PtAureo1a* KO strains.

After 12 d of RL incubation, the PtBLINDA1a protein expression in BlindA1a lines is similar to that of PtAUREO1a protein in the WT (Supplementary Fig. S6). Differences in gene expression change among samples were more profound after 10 min of BL exposure compared with the 60 min BL exposure (Fig. 4; Supplementary Fig. S7). The changes in gene expression in the BlindA1a mutants are mostly significantly different from A1aKO9, but similar to the WT. Eight genes (*PtAureo2*, *PtGPDH*, *PtJ38559*, *PtRitmo1b*, *PtLhcf15*, *PtdsCYC2*, *PtHSF3.2b*, and *PtDPH1*) exhibit a significant difference in BlindK32/K49 compared with the WT (Fig. 4). Notably, *PtLhcf15* is the only gene in BlindA1a lines with an expression pattern distinct from that of the WT and similar to that of A1aKO9 (Fig. 4D).

To test how the mutation in the PtBLINDA1a protein affects protein–DNA interaction, we performed a Y1H assay of PtBLINDA1a protein and the three promoter sites that strongly interact with PtAUREO1a: *PtdsCYC2-promo*, *PtAureo1a-promo*, and *PtAureo1c-promo* (Supplementary Fig. S8). PtBLINDA1a interacts with both *PtdsCYC2-promo* and *PtAureo1c-promo*, although yeast growth of PtBLINDA1a with *PtdsCYC2-promo* on the +AbA plate was much weaker than that of the A1a yeast strain (cf. Fig. 1B). There is no interaction between PtBLINDA1a and *PtAureo1a-promo* (Supplementary Fig. S8). Moreover, PtBLINDA1a fused with an additional yeast AD (PtBLINDA1a-AD) interacts with *PtdsCYC2-promo* but again not with *PtAureo1a-promo* or *PtAureo1c-promo* (Supplementary Fig. S8), which was a similar finding to that in the A1aAD yeast strain (cf. Supplementary Fig. S4), suggesting that having a yeast AD may hinder the interaction or the expression of the reporter gene (AbA resistance gene).

**Fig. 5.**
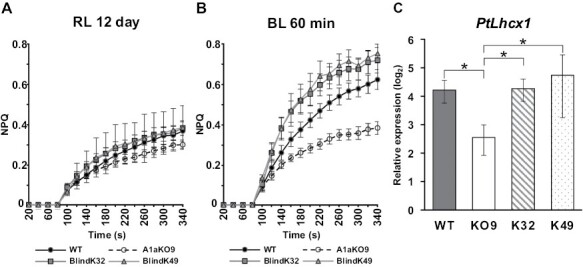
NPQ capacity in WT, A1aKO9, and BlindA1a mutants. (A and B) NPQ induction in the WT (solid line, filled circle), A1aKO9 (dashed line, open circle), BlindK32 (solid line, gray square), and BlindK49 (solid line, gray triangle) after 12 d of RL-acclimatized state (A) and after a shift to 60 min of blue light exposure (B). Mean values are shown with the SE (*n*=6 for WT, A1aKO9, BlindK32, and *n*=3 for BlindK49). (C) Relative gene expression of *PtLhcx1* in the WT (gray bar), A1aKO9 (KO9, white open bar), BlindK32 (K32, bar with diagonal stripes), and BlindK49 (K49, dotted bar) after 60 min of BL exposure. Mean values are shown with the SE (*n*=4 for the WT, KO9, and K32, and *n*=3 for K49). Statistically significant differences between KO9 and other samples were determined using a pair-wise fixed reallocation randomization test and marked with an asterisk (*P≤0.05).

In order to characterize BlindA1a mutants, we assessed their NPQ characteristics. After acclimation in RL for 12 d, NPQ capacities of all samples were very modest (<0.4 in all samples, Fig. 5A). After shifting cells to BL and exposing them for 60 min, WT and both BlindA1a mutants show a pronounced increase in NPQ capacity, whereas it remains low in A1aKO9, similar to the RL state (Fig. 5B). This result corresponds to the gene expression of *PtLhcx1*, a gene crucial for NPQ in *P. tricornutum* (Buck *et al.*, 2019): WT and both BlindA1a mutants display a significantly higher expression of the *PtLhcx1* gene than A1aKO9 after 60 min of BL exposure (Fig. 5C). Similarly, when samples are grown in white light (WL) conditions (35 µmol photons m^–2^ s^–1^ at 20 °C), both BlindK32 and BlindK49 display a similar NPQ capacity to the WT but different compared with A1aKO9 (Supplementary Fig. S9). A lower NPQ capacity of A1aKO9 than of the WT is consistent with previous reports (Serif *et al.*, 2017; Madhuri *et al.*, 2019) (Supplementary Fig. S9).

### Importance of functional PtAUREO1a for red light acclimation in *P. tricornutum*

**Fig. 6.**
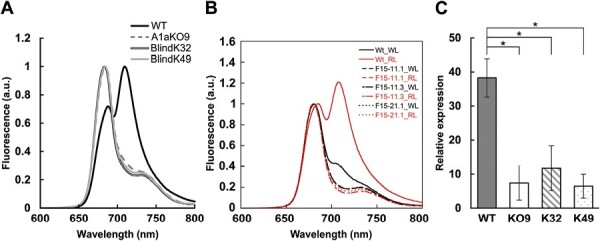
Expression of the red-shifted antenna complex in *P. tricornutum*. (A) Fluorescence emission spectra of WT (solid black line), A1aKO9 (dashed gray line), BlindK32 (solid double black line), and BlindK49 (solid light gray line) after 12 d of RL incubation. (B) Room temperature fluorescence emission spectra of WT and Lhcf15-KO lines grown under standard laboratory conditions in white light (WL), and after a shift to red light (RL) for 9 d. Cultures grown in WL are indicated with black lines, while those shifted to RL are indicated with red lines. (C) Real-time qPCR analysis of *Lhcf15* gene level in the WT (gray bar), A1aKO9 (KO9, white open bar), BlindK32 (K32, bar with diagonal stripes), and BlindK49 (K49, dotted bar) after 12 d of RL incubation. Mean values are shown with the SE (*n*=4 for the WT, KO9, and K32, and *n*=5 for K49). Statistically significant differences between the WT and other samples were determined using a pair-wise fixed reallocation randomization test and marked with an asterisk (*P≤0.05).

Despite similarities between the BlindA1a mutants and the WT, we identified a distinct phenotype in the BlindA1a mutants that differed from the WT yet closely resembled A1aKO9. While measuring the NPQ of RL-acclimatized samples, we observed that WT cells exhibit a stronger fluorescent emission than A1aKO9 and BlindA1a mutants, even though all samples were normalized to the same Chl *a* amount. Fluorescence emission spectra measured at room temperature after 12 d of RL cultivation in WT cells reveal two distinct peaks (Fig. 6): one at 688 nm, which is typical for PSII complexes, and the other at 710 nm, which indicates the presence of a specific red-shifted antenna bound to PSII (Herbstová *et al.*, 2015, 2017) (Fig. 6A). Based on biochemical purification followed by MS, this antenna had been assigned to the LHCF15 protein (Herbstová *et al.*, 2015). To verify this result, we created knockout strains of the *PtLhcf15* gene (Supplementary Fig. S10). The pronounced development of the red-shifted fluorescence maximum in the WT after RL cultivation, which is lacking in all Lhcf15-KO lines under both WL and RL conditions, proves that the PtLHCF15 protein is the source of the red-shifted antenna (Fig. 6B). This ultimately demonstrates that this antenna observed at the RL and transferring energy most likely to PSII is indeed exclusively provided by this single protein as proposed previously (Herbstová *et al.*, 2015, 2017). Interestingly, A1aKO9 and BlindA1a mutants do not show the development of the red-shifted antenna under RL conditions. Accordingly, the expression of the *PtLhcf15* gene in RL-cultivated A1aKO9 and BlindA1a mutants is significantly lower than that of the WT (Fig. 6C).

## Discussion

AUREOs are fascinating candidates for optogenetic tools as they are considered to be very fast and direct genetic regulators. PtAUREO1a, in particular, has been shown to play a significant role in overall gene regulation and to influence cell physiology upon light changes (Schellenberger Costa *et al.*, 2013; Mann *et al.*, 2020). However, previous studies tend to consider PtAUREO1a solely as a BL-dependent gene activator, and little is known about its function in the dark or RL, even though PtAUREO1a was demonstrated to bind to DNA in the dark (Banerjee *et al.*, 2016b; Heintz and Schlichting, 2016), and to distinctly affect gene expression in RL (Schellenberger Costa *et al.*, 2013; Mann *et al.*, 2020). Furthermore, despite the importance of the heterodimerization of bZIP TFs in gene regulation, hardly any dimer partners or co-regulators of PtAUREO1a are known.

Here, we conducted *in vitro* and *in vivo* experiments to elucidate the intricate molecular mechanisms underlying PtAUREO1a. We identified AUREO dimer partners as well as genes potentially regulated by PtAUREO1a. Moreover, we assess the different light absorption properties of PtAUREO paralogs and demonstrate how light perception by PtAUREO1a affects gene regulation *in vivo*.

### Genes directly regulated by PtAUREO1a are intermediate regulators

Interestingly, the six genes (*PtAureo1a*, *PtAureo1c*, *PtdsCYC2*, *PtbHLH2_PAS*, *PtHSF3.2b*, and *PtbZIP10*) whose promoter regions show interaction with PtAUREO1a are involved in light responses (Figs 1, 2; Supplementary Fig. S4). In addition, five of these genes encode TFs, with *PtdsCYC2* being the exception. *PtdsCYC2* has been identified as a regulator of cell division onset upon BL exposure after a period of darkness, under direct regulation by PtAUREO1a, PtbZIP10, or both (Huysman *et al.*, 2013). PtAUREO1a and PtAUREO1c are BL photoreceptors/TFs that regulate genes in response to light conditions (Kroth *et al.*, 2017). PtbHLH2_PAS is reported to regulate the Rubisco gene in response to fluctuating light (Matthijs *et al.*, 2017), while HSF3.2b is highly expressed under long-term fluctuating light conditions in *P. tricornutum* (Zhou *et al.*, 2022). It is highly plausible that the PtdsCYC2 protein and these five TFs may serve as intermediate regulators, orchestrating PtAUREO1a-initiated downstream signaling cascades to optimize photosynthesis and cellular metabolism for light acclimation.

### Four PtAUREO paralogs are cooperating partners

The functional dimerization of bZIP TFs is essential for their function (Ellenberger, 1994). Previous studies have made predictions of AUREO dimers using bioinformatics tools (Banerjee *et al.*, 2016b; Serif, 2017; Khamaru *et al.*, 2022b). Interestingly, possible dimer combinations within class 1 AUREO paralogs have been predicted in both *P. tricornutum* and the brown alga *Ectocarpus siliculosus*, while unstable dimerization was predicted between class 1 and class 2 AUREOs (Banerjee *et al.*, 2016b; Serif, 2017; Khamaru *et al.*, 2022b). However, previously, only three dimer combinations, PtAUREO1a–PtAUREO1a, PtAUREO1a–PtAUREO1c, and PtAUREO1a–PtbZIP10, have been experimentally confirmed *in vitro* (Huysman *et al.*, 2013; Banerjee *et al.*, 2016b).

In this study, we found that all 10 combinations of PtAUREO dimers can be formed in the absence of DNA using pull-down assays (Fig. 1A). Since DNA specificities of bZIP TFs may vary depending on dimer combinations (Amoutzias *et al.*, 2008; Rodríguez-Martínez *et al.*, 2017), different dimer combinations of PtAUREOs may bind to different stretches of DNA, thus broadening their biological roles *in vivo*.

As the synergetic activities of the PtAUREO1a and PtAUREO1c heterodimers have been proposed (Banerjee *et al.*, 2016b), we attempted to generate a yeast strain expressing both PtAUREO1a and PtAUREO1c; however, we failed to obtain a yeast strain expressing both proteins simultaneously.

Our pull-down assay results show that, depending on the protein interaction partners (GST–PtAUREOs), the eluted His6-PtAUREOs exhibit varying protein band intensities, which suggests that the protein–protein interaction affinities for each dimer combination are different. All of the His6-PtAUREO prey proteins that interacted with the GST–PtAUREO2 bait protein exhibited protein band intensities that were similar to, if not the same as, the input proteins (Fig. 1A). Contrary to previous predictions, this indicates a stable interaction between class 1 and 2 PtAUREOs, and also sheds light on non-light-induced class 2 PtAUREO regarding its potential role as a stabilizer of PtAUREO dimers.

### Each PtAUREO may have a distinct function

As all potential PtAUREO dimers are verified, understanding the feature and biological function of each PtAUREO is critical for understanding the cellular functions of individual PtAUREO dimers.

We found that full-length recombinant PtAUREOs have different light absorption properties (Fig. 3), suggesting that each PtAUREO might have a distinct function, which aligns with the notion proposed by Banerjee *et al.* (2016b), who suggested that individual PtAUREOs serve unique biological functions based on different diel transcript patterns of the four *PtAureo* genes. In addition, PtAUREO1b shows double peaks at the UV/A spectrum region, similar to PtAUREO1c, while PtAUREO1a exhibits a single peak (Fig. 3B). Indeed, PtAUREO1b and PtAUREO1c possess a serine residue (S303 and S199, respectively) displaying typical LOV1-like spectrum properties, while PtAUREO1a possesses a threonine (T255) within the LOV domain, thus displaying a LOV2-like pattern (Raffelberg *et al.*, 2013). The variability of this residue has an impact on the photocycle dynamics (Raffelberg *et al.*, 2013). In fact, PtAUREO1a and PtAUREO1c were reported to have different photocycle dynamics, and it was suggested that PtAUREO1a might function as a low-light sensor, and PtAUREO1c as a high-light sensor (Bannister *et al.*, 2019). Notably, both PtAUREO1b and 1c exhibit stronger absorption properties than PtAUREO1a, indicating a potentially higher sensitivity to dim light than PtAUREO1a, which may indicate that PtAUREO1b and 1c could act as low-light sensors. However, further verification is needed to compare the photocycles of all PtAUREOs using a small-angle X-ray scattering analysis described in Bannister *et al.* (2019).

Interestingly, our Y1H analyses revealed that each PtAUREO1a and PtAUREO1b interacts with gene promoter sites even in the absence of light (Fig. 1B). As we heterologously expressed PtAUREOs without an additional yeast AD in the yeast, our positive protein–DNA interaction results also demonstrate PtAUREO’s capability to initiate gene transcription. Therefore, while it has been experimentally confirmed that PtAUREO1a can bind to DNA in the dark *in vitro* (Banerjee *et al.*, 2016b; Heintz and Schlichting, 2016), we here demonstrate that both PtAUREO1a and PtAUREO1b can interact with DNA and initiate gene transcription in both dark and light conditions.

One more noticeable finding from Y1H assays is that although only a limited number of gene promoters were tested for the interaction with PtAUREO1b, we observed that PtAUREO1b interacted with the same promoters as PtAUREO1a (Fig. 1B). Furthermore, the interaction between PtAUREO1b and promoter sites appeared to be relatively strong, as the growth of A1b yeast was comparable with that of the positive control. Given that PtAUREO1a and PtAUREO1b have different light absorption properties, these results suggest that PtAUREO1b may serve a function distinct from that of PtAUREO1a while also supporting PtAUREO1a in certain circumstances, potentially including dark conditions.

When using proteins fused with the yeast AD, we observed that the Y1H assays, in some cases, yielded different results from those without the AD. For example, A1aAD protein does not interact with *PtAureo1a-* and *PtAureo1c-promo* (Supplementary Fig. S4), while these two promoters showed a strong interaction with PtAUREO1a protein alone (Fig. 1B). Also, A1aAD shows a strong interaction with *bZIP10-promo* (Supplementary Fig. S4D), which was not observed with PtAUREO1a (Fig. 2D). Similarly, when the yeast strain expresses the PtBLINDA1a protein alone, it interacts with the *PtAureo1c-promo*, while this interaction is no longer observed when AD is fused with the PtBLINDA1a (PtBLINDA1a-AD) (Supplementary Fig. S8).

These results indicate that potentially steric hindrances of conformational changes of the protein by the AD domain could be the cause. Despite ongoing debates on the conformational changes of AUREO protein, two notions seem to be consistent: (i) PtAUREO1a homo-dimerization can occur in the dark; and (ii) the conformational changes that occur in the LOV domain influence the bZIP domain’s DNA binding capacity (Herman *et al.*, 2013; Banerjee *et al.*, 2016b; Heintz and Schlichting, 2016).

Therefore, the fusion of the yeast AD at the C-terminus of PtAUREO1a, next to the LOV domain, might disturb the dimerization of LOV domains, or disturb the changes in structure of the flanking helices of the LOV domain (A’α and Jα), especially Jα, which is located at the C-terminus of the LOV domain. This suggests that although PtAUREO1a can function as a TF in the absence of light, its ability to regulate gene expression may depend on the flexibility of the LOV domain. Furthermore, our A1aAD data may indicate that PtAUREO1a in yeast—either as a homodimer or as a heterodimer with other yeast bZIP TFs—might function as a repressor (e.g. *bZIP10* gene), thus showing no interaction without an AD, and also that the transcription activation of a gene may be influenced by its sensitivity to DNA/protein binding affinity or binding structure.

### PtAUREO1a regulates *PtLhcf15* gene expression and is one of the critical elements for red light acclimation in the cell

Assessing the BlindA1a mutants shows both aspects of this aureochrome: its capacity to function as a TF without light stimulus, and the importance of the light sensory module for its functionality. Even though the PtBLINDA1a protein shows a considerably reduced light absorption compared with PtAUREO1a, it shows a similar functionality (Figs 4, 5). This suggests that the presence of the FMN in PtAUREO1a may not be necessary to fulfill many functions of the TF. It rather seems that the amount of bound FMN is necessary for PtAUREO1a for fine-tuning gene regulation.

According to Banerjee *et al*. (2016a), a single amino acid substitution in the LOV domain (V253M) results in an almost complete loss of FMN binding. However, our results reveal that full-length PtBLINDA1a retains FMN to some extent (Fig. 3), hence displaying a visible absorption peak at 446 nm. This might be because Banerjee *et al.* (2016a) only studied the LOV domain fragment, while we here investigated the full-length protein. A similar result was reported with *Arabidopsis* phot1, where a single amino acid substitution in the recombinant LOV2 domain results in an almost complete loss of light absorption, while only a subtle reduction of FMN binding (~10%) was observed when the same amino acid substitution was introduced to a full-length phot (Jones *et al.*, 2007). Therefore, it is plausible that PtBLINDA1a might retain some functionalities of WT PtAUREO1a.

Furthermore, as the PtAUREO proteins can dimerize, the reduced light absorption of PtBLINDA1a might have been compensated by the cooperation with other (light-absorbing) interacting partners, such as PtAUREO1b and 1c. Thereby, the interaction with those bZIP TFs may enable PtAUREO1a to effectively carry out its function and even respond to BL stimuli.

On the other hand, the different gene expression of *PtLhcf15* in A1aKO9 and BlindA1a mutants compared with the WT in RL conditions (Fig. 6C) not only supports previous observations that PtAUREO1a regulates genes without BL exposure (Schellenberger Costa *et al.*, 2013; Mann *et al.*, 2020), but also highlights the importance of an intact sensory module for the protein.

Furthermore, we demonstrate a novel connection between PtAUREO1a and the PtLHCF15 antenna protein (Fig. 6). It is known that *P. tricornutum* can shift light absorption up to ~700 nm to cope with the RL-enhanced conditions, resulting in an emission maximum of 710 nm. This red-shifted antenna complex comprises PtLHCF15 protein (Herbstová *et al.*, 2015). Our Lhcf15-KO data confirm that PtLHCF15 is crucial for red-shifted antenna expression and, thus, essential for adapting to RL-rich conditions. A recent report, which aligns with our data, has just emerged, demonstrating the importance of LHCF15 for longer wavelength light adaptation using *Lhcf15* knockout and complemented lines (Wang *et al.*, 2023). The authors further speculate that the Asn73 in helix B of LHCF15, in contrast to other light-harvesting proteins, may be the primary factor contributing to red-shifted absorption after the accumulation of LHCF15 (Wang *et al.*, 2023).

Since PtAUREO1a does not directly interact with *PtLhcf15-promo* (Fig. 2C), we suspect that another co-regulator is involved, such as the RL photoreceptor phytochrome (PtDPH1), which has been reported to mediate far-red light signaling (Fortunato *et al.*, 2016). Since gene expression of the *PtDPH1* gene is significantly affected by PtAUREO1a (Mann *et al.*, 2020), it is likely that RL acclimation is co-regulated by PtDPH1 and PtAUREO1a. Indeed, the gene expression of *PtDPH1* in KO9 and two BlindA1a lines, upon a shift from RL to BL, was highly up-regulated compared with the WT, which might be related to the absence or dysfunction of PtAUREO1a protein in KO9 and BlindA1a lines, respectively.

**Fig. 7.**
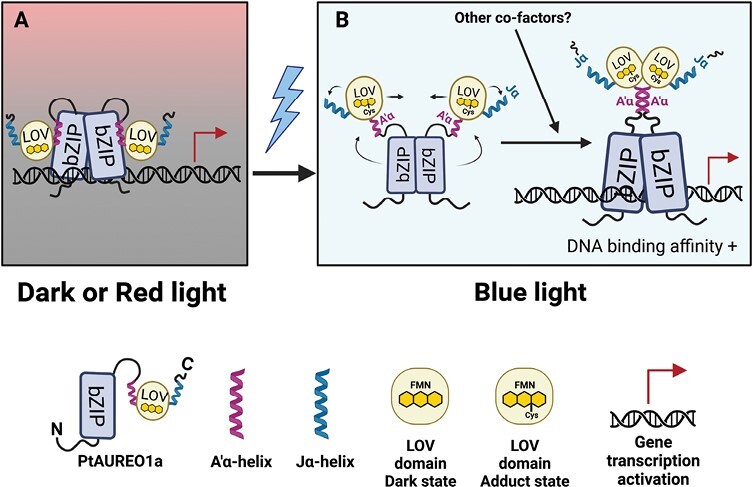
Simplified working model of PtAUREO1a’s functional mechanism under dark and blue light conditions. This working model is adapted from Goett-Zink *et al.* (2023) with modifications. (A) A PtAUREO1a dimer bound to the DNA in the absence of blue light illumination, most likely under dark or red light conditions. (B) Upon blue light illumination, a covalent adduct is formed between the FMN chromophore and a conserved Cys residue within the LOV domain (Herman *et al.*, 2013), followed by the unfolding of flanking helices, A'α and Jα. These structural changes lead to the dimerization of two LOV domains of PtAUREO1a. Consequently, the global conformational changes of PtAUREO1a increase the DNA binding affinity (Banerjee *et al.*, 2016b; Heintz and Schlichting, 2016). The image is created with BioRender.com.

As it is postulated that AUREOs evolved from a non-light-sensitive form to a light-sensitive form (Khamaru *et al.*, 2022a, Preprint), gene regulation of PtAUREO1a in the absence of BL illumination (RL or dark) might be indicative of its ancestry, and PtAUREO1a may have further evolved to serve comprehensive roles utilizing the sensory domain. Indeed, several studies have confirmed that the conformational changes of PtAUREO1a are influenced by BL illumination, specifically due to the changes in structure of the LOV domain (Herman *et al.*, 2013; Banerjee *et al.*, 2016b; Heintz and Schlichting, 2016; Goett-Zink *et al.*, 2023). Although it is confirmed that PtAUREO1a can dimerize in the dark (Banerjee *et al.*, 2016b; Heintz and Schlichting, 2016), BL induces the LOV domain to disassociate from the bZIP domain and induces a structural change of flanking helices of the LOV domain (Goett-Zink *et al.*, 2023) (Fig. 7). Specifically, it has been reported that under BL illumination, the Jα-helix influences the dimerizations of the bZIP domains, confirming that the bZIP dimer cannot be formed without this unfolding of the Jα-helix in BL conditions (Herman *et al.*, 2013; Goett-Zink *et al.*, 2023) and, further, the A'α-helix influences the LOV–LOV domain dimerization (Herman and Kottke, 2015; Goett-Zink *et al.*, 2023). Consequently, these structural changes in the LOV domain affect the bZIP domain’s flexibility and bZIP dimerization capacity, influencing PtAUREO1a’s gene regulation property.

Perhaps this might be the main reason for discrepancies between the WT and BlindA1a regarding certain gene expression changes upon RL to BL shift conditions. The reduced light absorption property of PtBLINDA1a protein might affect the flexibility of flanking helices, thereby possibly disrupting the dimerization with other bZIP protein partners under BL conditions.

However, it remains unclear how an intact LOV domain may contribute to gene regulation in RL. Although further biochemical and biophysical studies are needed to unravel how exactly PtAUREO1a initiates gene transcription in dark or RL conditions, our findings provide new insights into PtAUREO’s LOV domain, suggesting that the LOV domain in PtAUREOs may play a role in specific gene regulation in the absence of BL exposure (Fig. 7).

### Conclusion

**Fig. 8.**
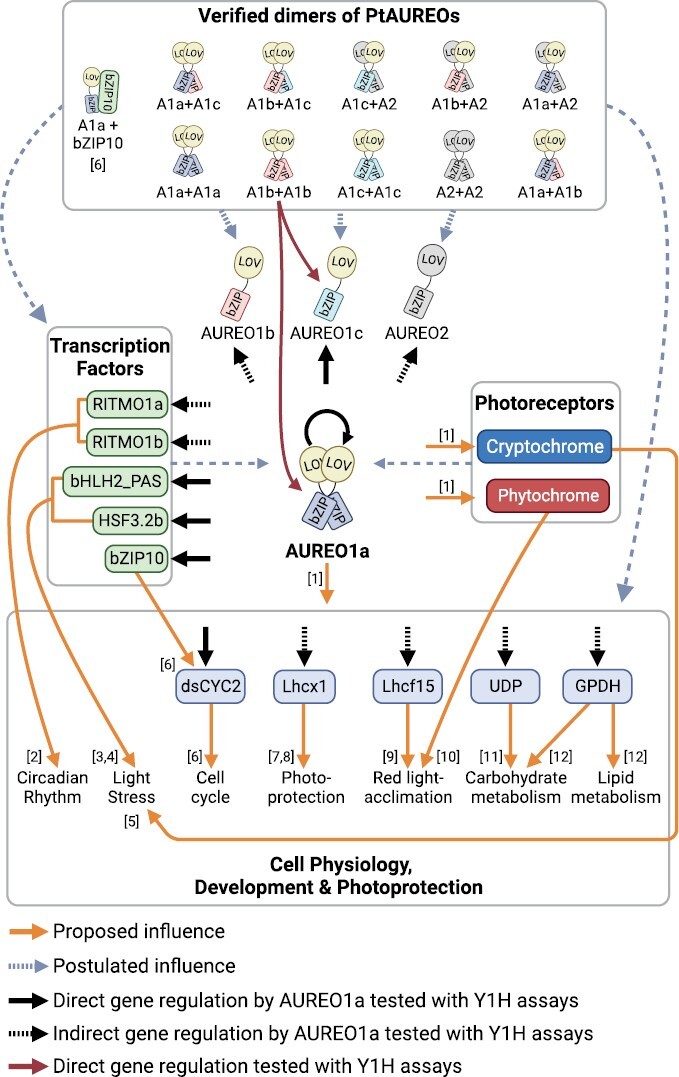
Hypothetical working model of overall gene regulation by PtAUREO1a. Each dimer combination is written with an abbreviation of proteins, PtAUREO1a, A1a; PtAUREO1b, A1b; PtAUREO1c, A1c; and PtAUREO2, A2. The references that were the primary source for the orange line arrow indicating ‘Proposed influence’ are listed with numbers: [1] Mann *et al.* (2020); [2] Annunziata *et al.* (2019); [3] Matthijs *et al.* (2017); [4] Zhou *et al.* (2022); [5] König *et al.* (2017a); [6] Huysman *et al.* (2013); [7] Buck *et al.* (2019); [8] Bailleul *et al.* (2010); [9] Herbstová *et al.* (2015); [10] Fortunato *et al.* (2016); [11] Kroth *et al.* (2008); [12] Yao *et al.* (2014). The image is created with BioRender.com.

PtAUREO1a is shown to be a potent regulator of gene expression. Given that a lack of PtAUREO1a cannot be complemented by other proteins (Mann *et al.*, 2020), it is evident that PtAUREO1a is an essential element that directly or indirectly controls TFs, photoreceptors, proteins, and genes to regulate overall gene expression (Fig. 8). Our findings suggest that not only PtAUREO1a, but also other PtAUREOs, may play more profound roles in gene regulation than is currently understood. Each PtAUREO may have a unique function with different light absorption properties, and potential dimerization of all PtAUREO may broaden their gene regulation roles *in vivo*.

Further investigations on the protein affinities of each PtAUREO dimer combination under different light conditions, and the identification of further binding motifs of each PtAUREO dimer utilizing protein–DNA identification assays such as ChIP-seq or DNA affinity purification sequencing (DAP-seq) would be very useful to understand PtAUREO’s biological functions and mechanisms, establishing a basis for future use of PtAUREOs as optogenetic tools.

While further experiments await elucidation of photoreactivity and DNA binding specificities of PtAUREOs, we here present a new view on PtAUREOs, in which these proteins are not mere photoreceptors that require BL to be activated, but rather TFs that have evolved utilizing BL illumination for sophisticated gene regulation. Based on the findings of this study, PtAUREOs are anticipated to be utilized as powerful natural optogenetic tools that can finely regulate genes under desired conditions, including dark and specific wavelengths of light.

## Supplementary data

The following supplementary data are available at *JXB* online.

Fig. S1. Coomassie blue stain gel image of GST, and GST- or His6-tagged recombinant PtAUREOs.

Fig. S2. Screening of yeast strains transformed with the respective *PtAureo* genes.

Fig. S3. Yeast viability test under continuous blue light conditions.

Fig. S4. Interaction assay of PtAUREO1a-AD and the promoter sites of the respective genes.

Fig. S5. Screening of BlindA1a mutants.

Fig. S6. PtAUREO1a and PtBLINDA1a protein expression in the WT and BlindA1a lines after 12 d of red light incubation.

Fig. S7. Gene expression changes of selected genes after a shift from 12 d of red light incubation to 60 min of short blue light exposure.

Fig. S8. The interaction assay of PtBLINDA1a and PtBLINDA1a-AD with the promoter sites that showed a strong interaction with PtAUREO1a.

Fig. S9. Non-photochemical quenching (NPQ) capacity in wild-type (WT), A1aKO9, and BlindA1a mutants.

Fig. S10. Characterization of Lhcf15 knockout (Lhcf15-KO) lines.

Table S1. Plasmids used for Y1H assays.

Table S2. Primers used for Y1H assays.

Table S3. Primers used for the generation and verification of BlindA1a and Lhcf15-KO mutants.

Table S4. Primers used for qPCR analyses.

Table S5. Expected molecular weight of His6- or GST-tagged recombinant PtAUREO proteins.

Table S6. Genes that are highly affected by PtAUREO1a.

erad478_suppl_Supplementary_Tables_S1-S6_Figures_S1-S10

## Data Availability

All the data supporting our findings are available within the manuscript and the supplementary data.

## Abbreviations

A1a: AUREO1a
A1b: AUREO1b
A1c: AUREO1c
A2: AUREO2
AbA: aureobasidin A
Aureo: aureochrome gene
AUREO: aureochrome protein
BL: blue light
bZIP: basic leucine zipper
GPDH: glyceraldehyde phosphate dehydrogenase
GST: glutathione *S*-transferase
HSF: heat shock factor
LOV: light-, oxygen-, voltage-sensing
NPQ: non-photochemical quenching
phot: phototropin
RL: red light
SD: synthetic-defined
TF: transcription factor
UDP: UDP-glucose-pyrophosphorylase
Y1H: yeast one-hybrid
