# Supplementary material for: Identification of promoter targets by Aureochrome 1a in the diatom Phaeodactylum tricornutum
Source: J Exp Bot. 2023 Dec 8;75(7):1834–51. doi: 10.1093/jxb/erad478 (PMC10967249; doi:10.1093/jxb/erad478)
Supplement: erad478_suppl_Supplementary_Tables_S1-S6_Figures_S1-S10 [file erad478_suppl_supplementary_tables_s1-s6_figures_s1-s10.pdf]

Supplementary data

Identification of promoter targets by Aureochrome 1a in the diatom *Phaeodactylum tricornutum*

Soo Hyun Im [1,*], Bernard Lepetit [1,5], Niccolò Mosesso [2], Sandeep Shrestha [1], Laura Weiss [1], Marianne Nymark [3], Robert Roellig [4], Christian Wilhelm [4], Erika Isono [2], and Peter G. Kroth [1,*]

Supplementary Figures

Supplementary Fig. S1

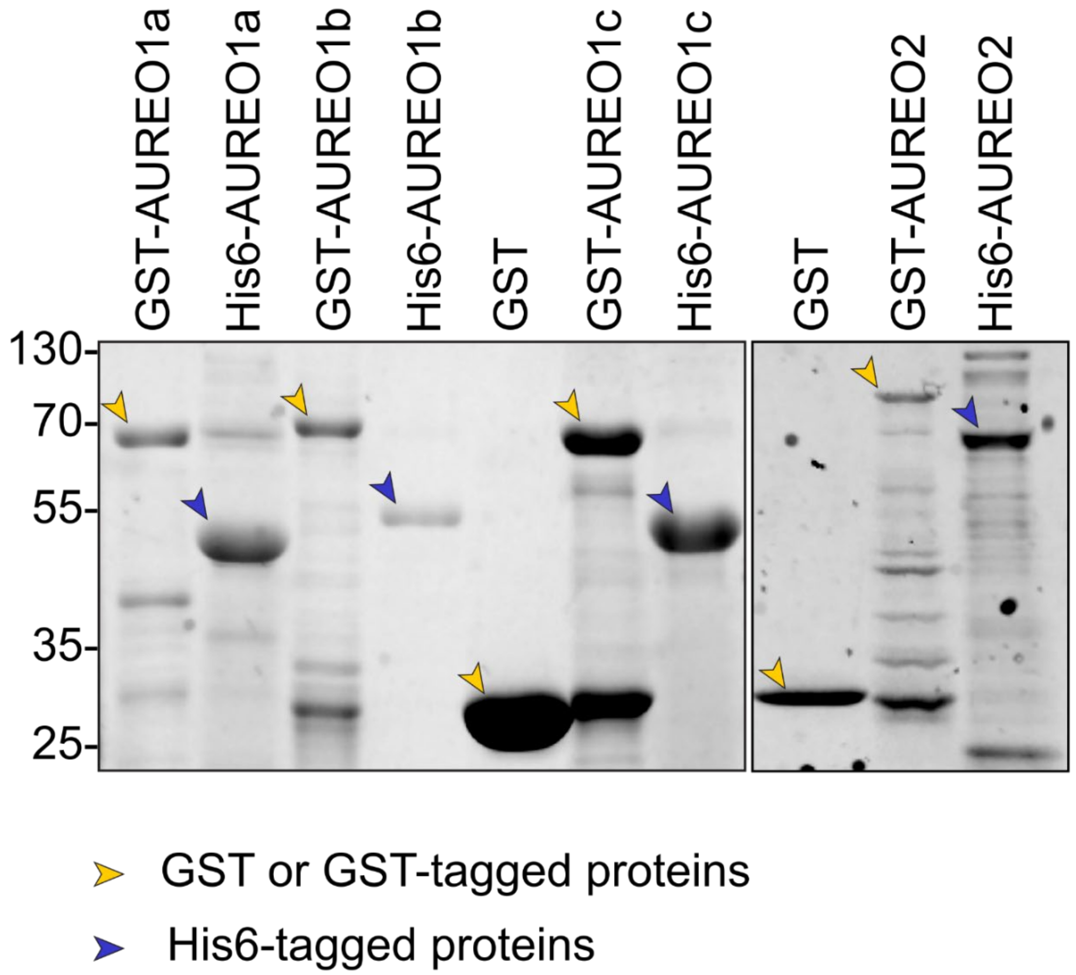

Supplementary Fig. S1. Coomassie blue stain gel image of GST, and GST- or His6-tagged recombinant PtAUREOs.

Supplementary Fig. S2

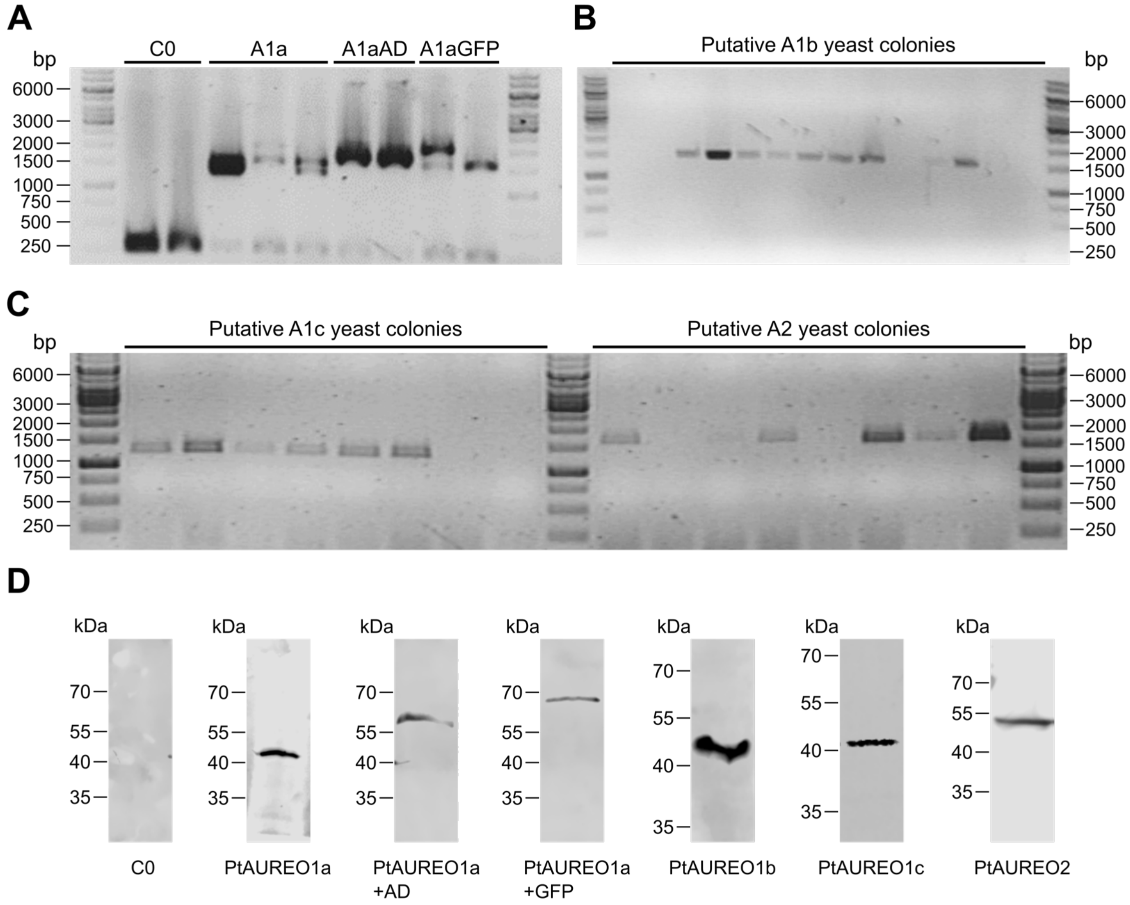

Supplementary Fig. S2. Screening of yeast strains transformed with the respective *PtAureo* genes. Yeast colony PCR was performed to verify the transformation of the V1 constructs in the respective yeast strains. (A), Yeast colony PCR with the putative yeast strains integrated with an empty vector V1 (C0), with a V1Aureo1a (A1a), with a V1Aureo1a-AD (A1aAD), and with a V1Aureo1a-GFP (A1aGFP). (B), Yeast colony PCR with the putative A1b yeast strains. (C), Yeast colony PCR with the putative A1c yeast and A2 yeast strains. (D), Western blot of each yeast strain using the respective anti-PtAUREO-specific antisera.

Supplementary Fig. S3

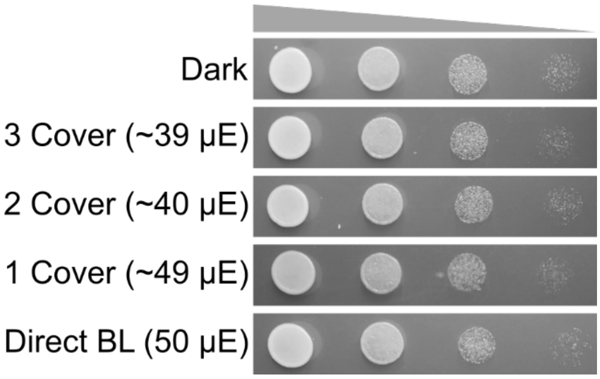

Supplementary Fig. S3. Yeast viability test under continuous blue light conditions. Yeast cells were spotted on YPDA plates and grown in dark and different intensities of blue light conditions (max. 50 μmol photons m^{-2} s^{-1}) for three days.

Supplementary Fig. S4

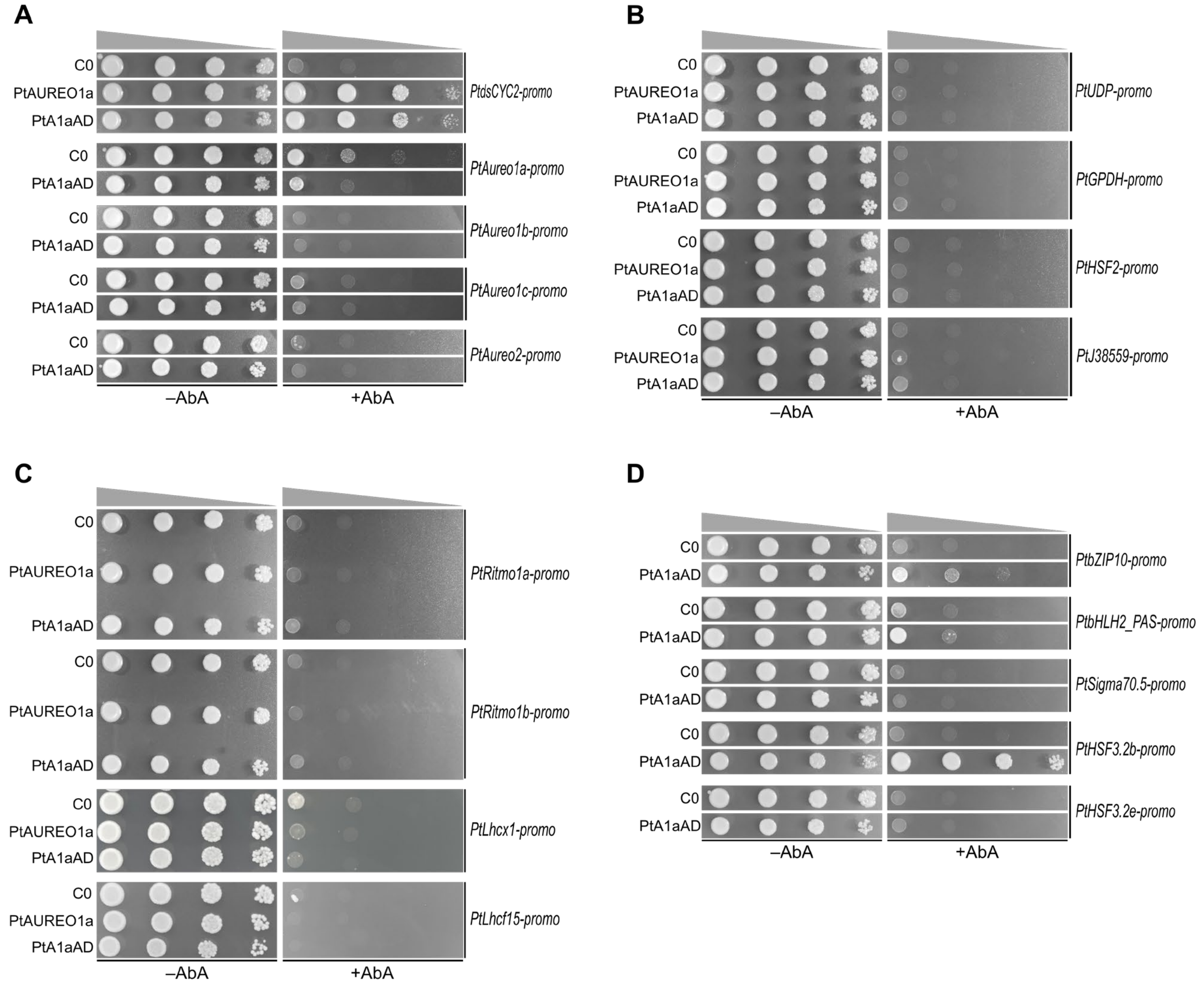

Supplementary Fig. S4. Interaction assay of PtAUREO1a-AD and the promoter sites of the respective genes. The names of the proteins expressed in yeast strains are listed on the left, and the gene promoter names are listed on the right (*gene name-promo*). C0 is a negative control, a yeast strain with the respective promoter site without any PtAUREO protein expression. Yeast strains were spotted in serial dilution on SD/–Leu/–Ura plates with or without AbA antibiotic (+AbA or –AbA). Cell growth on the +AbA plate indicates the interaction between the respective PtAUREO and the promoter site. Every interaction assay was compared to the negative control to verify the protein-DNA interaction, and all interaction assays were performed independently at least twice. Interaction between PtAUREO1a and *PtdsCYC2-promo* is used as a positive control. (A–D), The promoter sites of (A), *PtAureo* genes; (B), genes that are highly affected by PtAUREO1a; (C), genes that are responsible for a specific physiological feature; (D), selected transcription factor genes. For (B) and (C), C0, A1a, and A1aAD yeast strains with respective gene promoter sites were spotted on the same plate. Therefore, the non-cropped image is presented to show that negative controls for each gene promoter site on (B) and (C) are the same image as Fig. 2B and 2C.

Supplementary Fig. S5

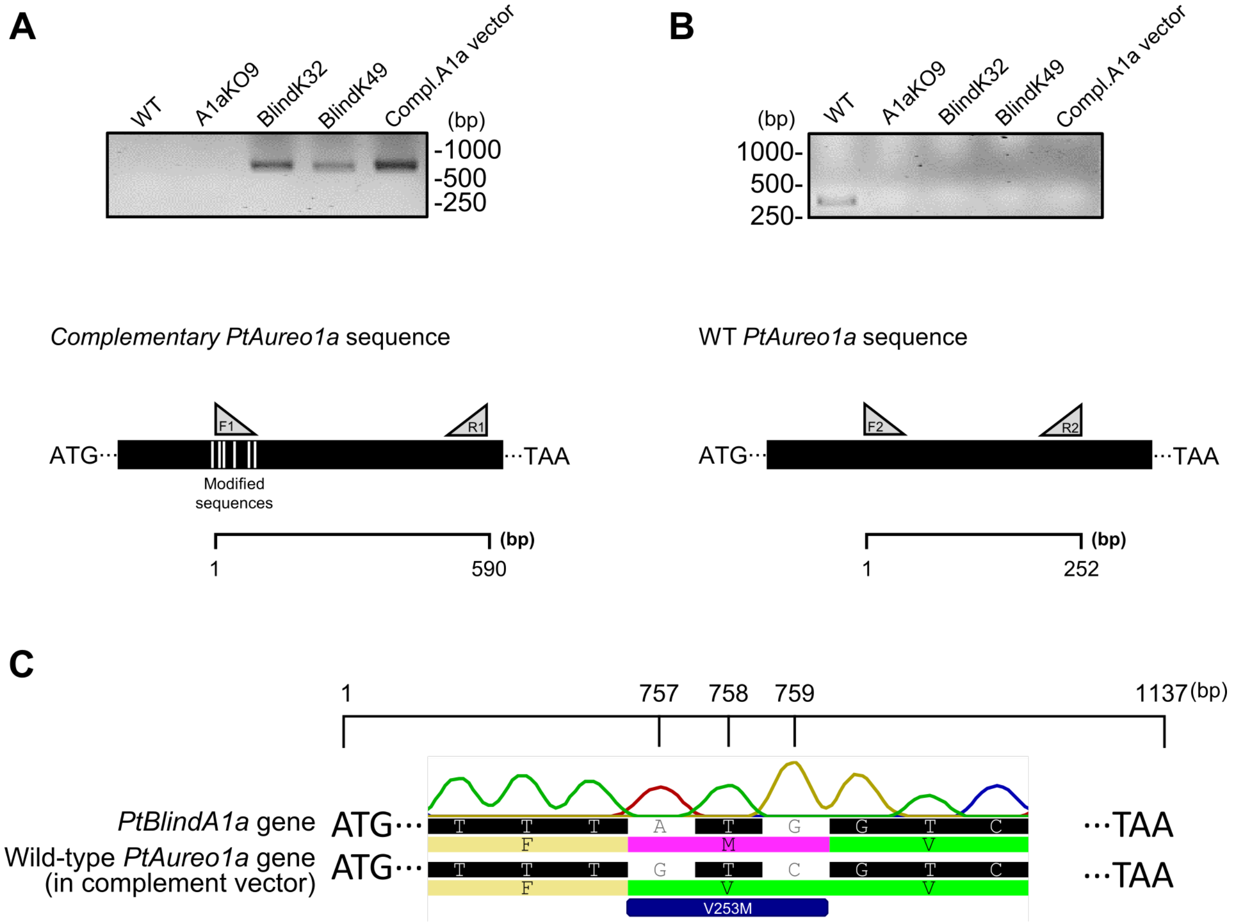

Supplementary Fig. S5. Screening of BlindA1a mutants. (A, B), PCR result of WT, A1aKO9, and two BlindA1a mutants using the complementary *PtAureo1a* gene-specific primers (A), and the wild-type *PtAureo1a* gene-specific primers (B). Schemes of primer binding sites for the respective primer pair are shown below the PCR pictures. (C), Sanger sequencing result of the *PtBlindA1a* gene in BlindK32 and BlindK49.

Supplementary Fig. S6

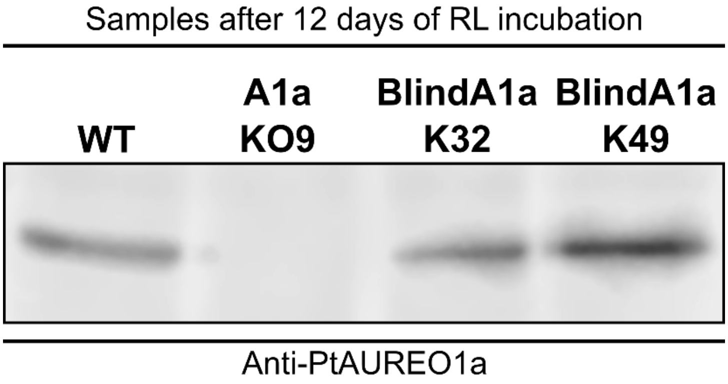

Supplementary Fig. S6. PtAUREO1a and PtBLINDA1a protein expression in the WT and BlindA1a lines, respectively, after 12 days of red light incubation. Samples were incubated under red light (RL) conditions for 12 days, and the same concentration of total protein from each sample (40 µg) was loaded on the gel to analyze protein levels. The western blot was performed with three independent replicates, and one representative result is shown.

Supplementary Fig. S7

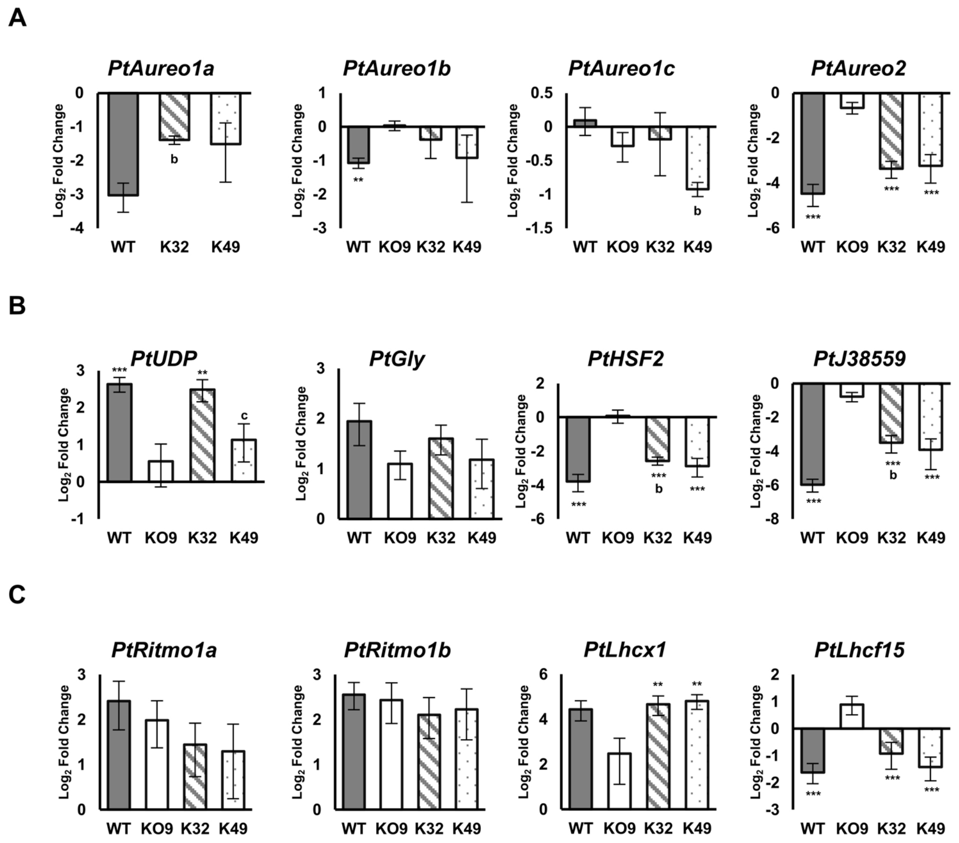

Supplementary Fig. S7. Gene expression changes of selected genes after a shift from 12 days of red light incubation to 60 min of short blue light exposure. Mean values of WT (grey bar), A1aKO9 (KO9, white hollow bar), BlindK32 (K32, bar with diagonal stripes), and BlindK49 (K49, dotted bar) are shown with standard error (n = 4 for WT, KO9, K32, and n = 3 for K49). Statistically significant differences between samples were determined using a pair-wise fixed reallocation randomization test using REST. Values that are significantly different compared to KO9 were marked with asterisks (**: $p \leq 0.01$, and ***: $p \leq 0.005$). Values that are significantly different compared to WT are marked with the letters (b: $p \leq 0.01$, and c: $p \leq 0.005$). (A), Four *PtAureo* genes. (B), Genes that are highly affected by PtAUREO1a. (C), Genes that are responsible for a specific physiological feature.

Supplementary Fig. S8

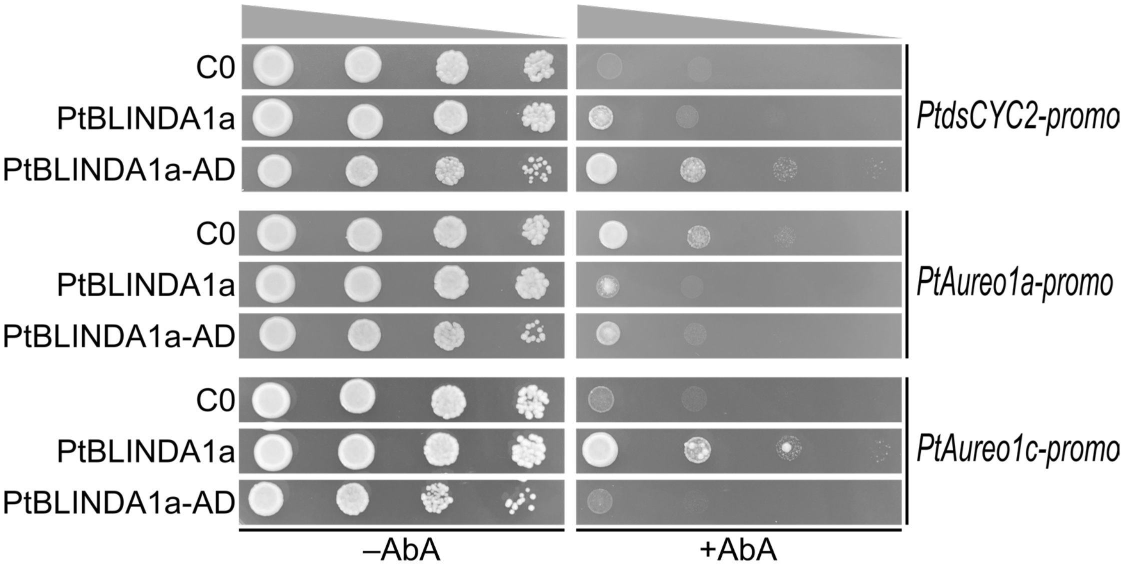

Supplementary Fig. S8. The interaction assay of PtBLINDA1a and PtBLINDA1a-AD, respectively, with the promoter sites that showed a strong interaction with PtAUREO1a. The names of the proteins expressed in yeast strains are listed on the left, and the gene promoter names are listed on the right. Yeast strains were spotted in serial dilution on SD/–Leu/–Ura plates with or without AbA antibiotic (+AbA or –AbA). Cell growth on the +AbA plate indicates protein-DNA interaction. C0 is a negative control, a yeast strain with the respective promoter site without any PtAUREO protein expression. Every interaction assay was compared to the negative control to verify the protein-DNA interaction.

Supplementary Fig. S9

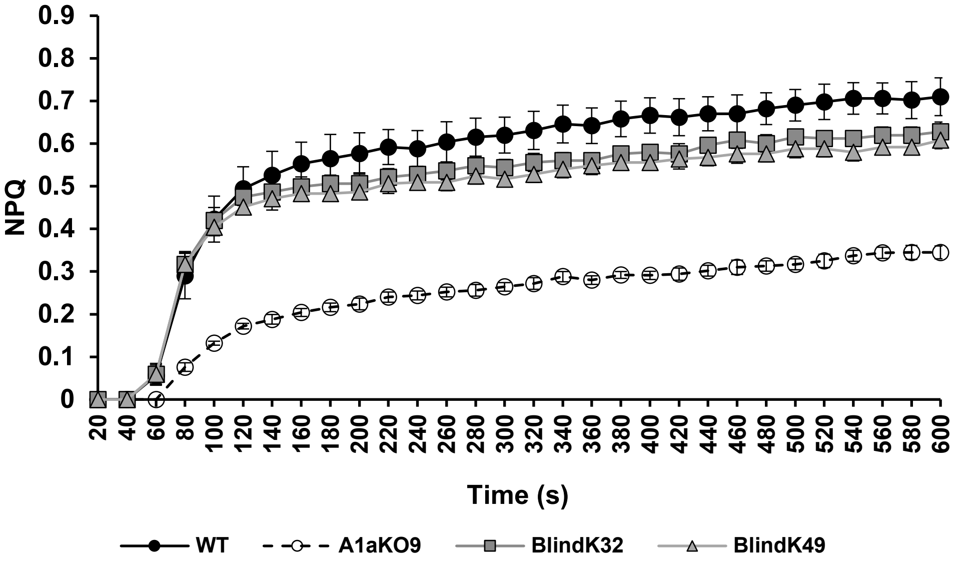

Supplementary Fig. S9. Non-photochemical quenching (NPQ) capacity in wild-type (WT), A1aKO9, and BlindA1a mutants. NPQ induction of WT (solid line, black circle), A1aKO9 (dashed line, hollow circle), BlindK32 (solid line, grey square), and BlindK49 (solid line, grey triangle) when grown under white-light conditions (35 µmol photons m^{-2} s^{-1} at 20 °C). Mean values are shown with standard error (n = 4).

Supplementary Fig. S10

A

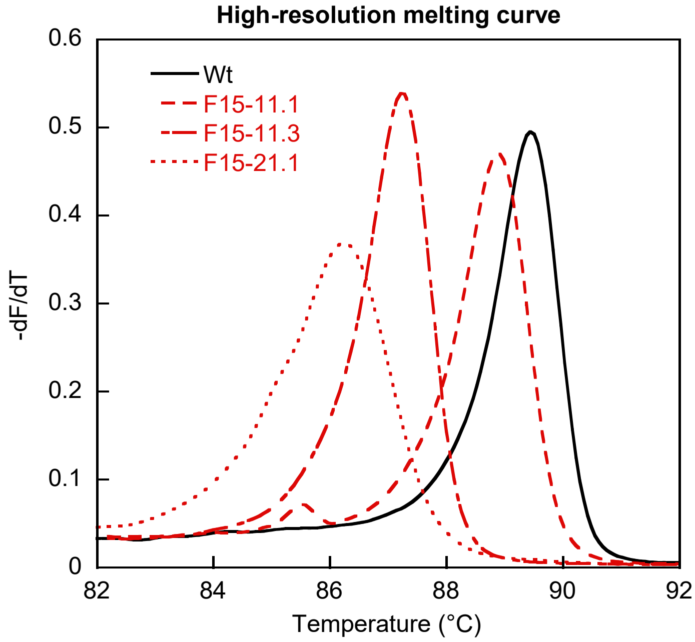

B

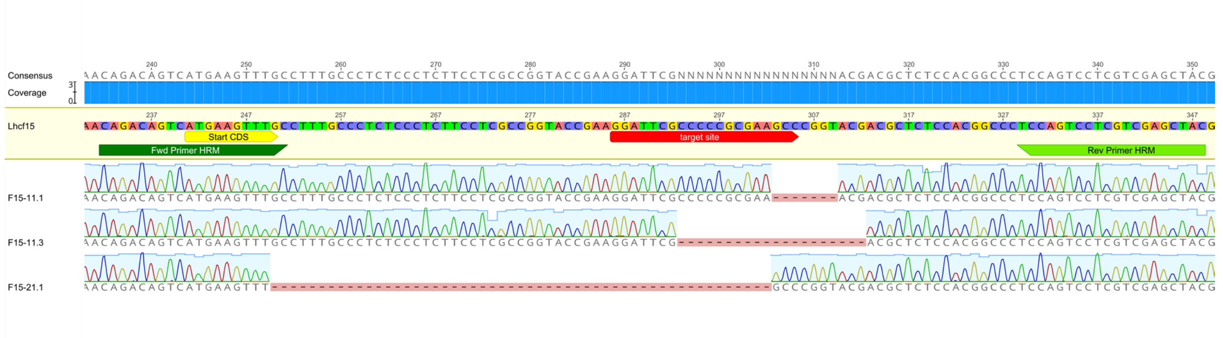

Supplementary Fig. S10. Characterization of Lhcf15 knock-out (Lhcf15-KO) lines. (A), High-resolution melting curve analysis of three different Lhcf15-KO lines generated by CRISPR-Cas gene editing. The shifted melting peaks indicate the altered genetic sequence. (B), Sequencing result of the three KO lines. The target sequence chosen for the sgRNA design is indicated in red. Primers for performing the PCR used for the high-resolution melting curve analysis in (A) are indicated in dark and light green. Lhcf15-11.1 contains a 7 bp deletion, Lhcf15-11.3 a 20 bp deletion, and Lhcf15-21.1 a 53 bp deletion. No wild-type (WT) allele could be sequenced in these lines, indicating either a homogeneous mutation of both alleles in all three lines, or a huge insertion or deletion, potentially involving a chromosomal rearrangement of the mutated allele.

Supplementary Tables

Supplementary Table S1: Plasmids used for Y1H assays

Plasmid name	Further termed	Information
p405ADH1	V1	Yeast expression vector for expressing the protein of interest without a yeast activation domain
pGADT7	pGADT7	Yeast expression vector for expressing the protein of interest fused with a yeast activation domain
pAbAi	V2	Yeast reporter vector

Supplementary Table S2: Primers used for Y1H assays

Oligo name	Sequence (5' to 3')	Application
Aureo1a_Fwd_XbaI	tggaactctagaATGACCGACAACAACAAGAGC	Amplification of *PtAureo1a* gene for integration into the p405ADH1 vector (V1)
Aureo1a_Rev_SalI	tggaacgtcgacTTAGTCTTCGTCATCGTTGGC	
Aureo1a_Rev2_BamHI	tggaacggatccTGTCTTCGTCATCGTTGGCC	Reverse primer for amplification of *PtAureo1a* gene with BamHI recognition sequence. This primer was paired with the Aureo1a_Fwd_XbaI forward primer
Gal4AD_Fwd_BamHI	tggaacggatccAGAGAAAGGTCGAATTGGGTAC	Amplification of GAL4 AD sequence from pGADT7 vector (V2)
Gal4AD_Rev_SalI	tggaacgtcgacTTAGCTCGCCCTATAGTGAGTCG	
GFP_Rev_SalI	tggaacgtcgacTTACTTGTACAGCTCGTCCATG	Reverse primer for amplification of *PtAureo1a-GFP* gene for integration into V1. This primer was paired with the Aureo1a_Fwd_XbaI forward primer. *PtAureo1a-GFP* gene was amplified from a pPha-T1-Aureo1a-GFP vector described in Schellenberger Costa *et al.* (2013).
Aureo1b_Fwd	**gctggccgctctagaactagtggatcccccc**ATGGATGATTTTGATTTGAACG	Amplification of *PtAureo1b* gene for integration into V1
Aureo1b_Rev	**gataagcttgatatcgaattcctgcagccc**TCACTCGCTACTATCATCTTTTG	
Aureo1c_Fwd	**gctggccgctctagaactagtggatcccccc**ATGGCGGACCAAGCCAAG	Amplification of *PtAureo1c* gene for integration into V1
Aureo1c_Rev	**gataagcttgatatcgaattcctgcagccc**CTAACTACTAGTAGTGGTCACGGG	
Aureo2_Fwd	**gctggccgctctagaactagtggatcccccc**ATGGCCCAAAATTTGCAAATG	Amplification of *PtAureo2* gene for integration into V1
Aureo2_Rev	**gataagcttgatatcgaattcctgcagccc**CTACGACGATCTACCTGCG	
Seq_V1_pADH1_Fwd	TGTTTCCTCGTCATTGTTCTCG	Sequencing primers used for V1 constructs verification
Seq_V1_tCYC1_Rev	GGGACCTAGACTTCAGGTTGTC	
Seq_V2_Fwd	AGTCTGTGCTCCTTCCTTCG	Sequencing primers used for V2 constructs verification
Seq_V2_Aur1C_Rev	CCTTCAACAACGTTTGATGGGG	
A1a promoter_Fwd_ Hind3	cccaagcttAGCTCGACAGTGACGAATTC	Amplification of the promoter site of *PtAureo1a* gene for integration into V2
A1a promoter_Rev_XmaI	gggcccgggGCGTGAACATGACGTCTCC	
A1b promoter_Fwd_ Hind3	cccaagcttAGCAGGTGAGAAGTGCGTAC	Amplification of the promoter site of *PtAureo1b* gene for integration into V2
A1b promoter_Rev_ XmaI	gggcccgggTCGTTCAAATCGAAATCATCCATC	
A1c promoter_Fwd_ Hind3	cccaagcttCTGTAGCCTTCACGACGC	Amplification of the promoter site of *PtAureo1c* gene for integration into V2
A1c promoter_Rev_XmaI	gggcccgggTGGTGTGTATGTGTGTGGAC	
A2 promoter_Fwd_ Hind3	cccaagcttAACGAGAGATCACGGGAAC	Amplification of the promoter site of *PtAureo2* gene for integration into V2
A2 promoter_Rev_ XmaI	cccccccgggTGGTGAAATTGAAGTCGAGGG	
dsCYC2 promoter_Fwd_Hind3	cccaagcttGCATGGACTCGAATAGAATATGTGG	Amplification of the promoter site of *PtdsCYC2* gene for integration into V2
dsCYC2 promoter_Rev_KpnI	cggggtaccGACGTTATAGTTAATCAGTATTTCAGG	

RITMO1a promoter_Fwd_Hind3	cccaagcttCTAGCGTTGTTTGAGCGAGC	Amplification of the promoter site of *PtRitmo1a* gene for integration into V2
RITMO1a promoter_Rev_XmaI	gggcccgggAATTGTATTCGGTCAGCTACGAC	
RITMO1b promoter_Fwd_Hind3	cccaagcttTTCCGAACCGTAGCGATTG	Amplification of the promoter site of *PtRitmo1b* gene for integration into V2
RITMO1b promoter_Rev_XmaI	gggcccgggCTATTCCTGTGCAACGTTCG	
UDP promoter_Fwd_Hind3	cccaagcttCGAGACACAACGGGATCAAC	Amplification of the promoter site of *PtUDP* gene for integration into V2
UDP promoter_Rev_KpnI	cggggtaccGAAGGAACGCAAATTCACAGTG	
GPDH promoter_Fwd_Hind3	cccaagcttCCGGTCAATCAGCATGTCG	Amplification of the promoter site of *PtGPDH* gene for integration into V2
GPDH promoter_Rev_XmaI	cccccccgggGGTCGTTTTGTGAGTGCGG	
HSF2 promoter_Fwd_Hind3	cccaagcttTGTCAATCGAATTTGTCTGGC	Amplification of the promoter site of *PtHSF2* gene for integration into V2
HSF2 promoter_Rev_XmaI	tcccccccgggTCATCATTTTGGACGTTGCG	
38559 promoter_Fwd_Hind3	cccaagcttGACCAAACCATCCTGCTC	Amplification of the promoter site of *PtJ38559* gene for integration into V2
38559 promoter_Rev_XmaI	tcccccccgggCCCACCTTTATCTTCATGTTG	
bZIP10 promoter_Fwd_Hind3	cccaagcttGCTGACAATCTTGCCGGAG	Amplification of the promoter site of *PtbZIP10* gene for integration into V2
bZIP10 promoter_Rev_XmaI	tcccccccgggGGGGGATCGTTTGTTCGC	
bHLH2_PAS promoter_Fwd_SacI	tcccgagctcGGTGGTCTTCAATGAGGTC	Amplification of the promoter site of *PtbHLH2_PAS* gene for integration into V2
bHLH2_PAS promoter_Rev_XmaI	tcccccccgggAGTTGAATTTGAGGCGACG	
Sigma70.5 promoter_Fwd_ SacI	tcccgagctcGTGTGACGTTCGATTCCTCTC	Amplification of the promoter site of *PtSigma70.5* gene for integration into V2
Sigma70.5 promoter_Rev_ XmaI	tcccccccgggTCAATGTGACGTTTGCTAGCG	
HSF3.2b promoter_Fwd_SacI	tcccgagctcGCCGTTCACATCGTCTTAATG	Amplification of the promoter site of *PtHSF3.2b* gene for integration into V2
HSF3.2b promoter_Rev_XmaI	tcccccccgggAGAAGAGCAACGAAGAGACTAC	
HSF3.2e promoter_Fwd_SacI	tcccgagctcCAGATCGCTTTAAACGCCTATG	Amplification of the promoter site of *PtHSF3.2e* gene for integration into V2
HSF3.2e promoter_Rev_XmaI	tcccccccgggGTGAAGTCGAACACCAATACAG	

The primer overhangs are written in lowercase letters. The respective restriction enzyme recognition sequences are underlined. As *PtAureo1b*, *PtAureo1c*, and *PtAureo2* genes were integrated into V1 via homologous recombination, each primer contains flanking sequences that are homologous to the V1, and are written in bold lowercase letters.

Supplementary Table S3: Primers used for the generation and verification of BlindA1a and Lhcf15-KO mutants

Oligo name	Sequence (5' to 3')	Application
V253M_F	CAGCAAAACTTTGTCGTCACCG	Site-direct mutagenesis of *PtAureo1a* gene
V253M_R	CGCCGTTTGGAGGGCCTT	
BlindA1a Seq F	GGACGTTGATGGACTCCTGG	Sequencing for BlindA1a_V253M plasmid
BlindA1a Seq R	CGTCAAGTTGAGAAAGCCCTG	
SM25 F	AACCCATCACTTGCAGTACGC	Verification of wild-type *PtAureo1a* gene
SM68 R	TCGCCCAAGTGCGAACG	
SM22	TCGCTTTTGAAAGAAGAAAATG	Verification of BlindA1a_V253M plasmid transformation
Aureo1a_Rev_SalI	TGGAACGTCGACTTAGTCTTCGTCATCGTTGGC	
Lhcf15_forward	CAGACAGTCATGAAGTTTGC	Verification of Lhcf15-KO using a high-resolution melting curve analysis
Lhcf15_reverse	GTAGCTCGACGAGGACTGGA	

Supplementary Table S4: Primers used for qPCR analyses

Oligo name	Sequence (5' to 3')	Application
Phatr3_J47943 F	ATGCCGACACAGTCTACCG	Reference gene for the qPCR analysis
Phatr3_J47943 R	GGAGAGGTGGTTGTTGTTGC	
UDP qPCR F	GTCTCGTCTCGGGTGATTCC	qPCR for *PtUDP*
UDP qPCR R	CGTCCCCCTTGACTGGCAA	
GPDH qPCR F	TCATCGGTAGCGGTAACTGG	qPCR for *PtGPDH*
GPDH qPCR R	ACCTGCGATTCGAAAAAGGG	
HSF2 qPCR F	ATGACTTACGCATCTCCCAATA	qPCR for *PtHSF2*
HSF2 qPCR R	GGGTCGGTCAGTAGTTTTCGAG	
38559 qPCR F	TACTTGGAACGTCTCGGTCG	qPCR for *PtJ38559*
38559 qPCR R	TAGAAAGCGGTTGCCAAGAC	
Aureo1a qPCR F	CCACCACCACCAACACTAGG	qPCR for *PtAureo1a*
Aureo1a qPCR R	AGGCGTGAACATGACGTCT	
Aureo1b qPCR F	GCTGTACCTCGAGTCCGTTG	qPCR for *PtAureo1b*
Aureo1b qPCR R	AAGCATTGCTGTCCCATTGTC	
Aureo1c qPCR F	ACGTTGCGTCCACACACATA	qPCR for *PtAureo1c*
Aureo1c qPCR R	GTCGTCGAGATCCAGAGTGG	
Aureo2 qPCR F	GTTCTCAAGGAGCTGGTCAAG	qPCR for *PtAureo2*
Aureo2 qPCR R	AACGAGTGTTGGGAACTCTGG	
Lhcx1 qPCR F	CAAACAAGCCGTGCGTC	qPCR for *PtLhcx1*
Lhcx1 qPCR R	CGAATCCAAGAGGGTCGAA	
Ritmo1a qPCR F	CGACCATGAGTGATTTTCAGTTT	qPCR for *PtRitmo1a*
Ritmo1a qPCR R	ACGTGGACGATTCCGAACC	

Ritmo1b qPCR F	GCAATTGCCTGTAAACACGGT	qPCR for *PtRitmo1b*
Ritmo1b qPCR R	TGTTGTTGACTATTGATGGCTCC	
Lhcf15 qPCR F	CGAGCAACATTGACGAAG	qPCR for *PtLhcf15*
Lhcf15 qPCR R	GACGGAATAGCATTGACG	
bZIP10 qPCR F	GTGCCGCGGCTTTAACTATG	qPCR for *PtbZIP10*
bZIP10 qPCR R	TGACACCTGGGATGGATTGC	
dsCYC2 qPCR F	ACGAATGTACTGGTCTCTGCTG	qPCR for *PtdsCYC2*
dsCYC2 qPCR R	AGCCAATCCCACGAGTGAAG	
bHLH2_PAS qPCR_ F	GGCAACTGCTATTCGGCATG	qPCR for *PtbHLH2_PAS*
bHLH2_PAS qPCR_ R	GGATCCGCCTGAAACGAAG	
HSF3.2b qPCR F	CTGGTGTTGAAACTGCGTCG	qPCR for *PtHSF3.2b*
HSF3.2b qPCR R	TGCTGCGGAAGTACAAGCG	
DPH1 qPCR F	GCTGTAGCCAAACCGCATATTG	qPCR for *PtDPH1*
DPH1 qPCR R	AGCAACCCCCACAGATCATTG	

Supplementary Table S5: Expected molecular weight of His6- or GST-tagged recombinant PtAUREO proteins

Protein	Size (kDa)	
	His6-tagged	GST-tagged
PtAUREO1a	43.7	68.3
PtAUREO1b	48.9	73.5
PtAUREO1c	38	62.6
PtAUREO2	52.7	75.7

Supplementary Table S6: Genes that are highly affected by PtAUREO1a. Expression fold change levels were taken from Mann *et al.* (2020). The promoter sites of selected genes were used for the Y1H interaction assays

Group	GeneID	Gene name	Abbreviation	Log_2-fold gene expression fold change (BL 10 min/ RL)	
				WT	A1aKO mutant
	Phatr3_J34956	*PtdsCYC2*	*PtdsCYC2*	1.47	2.29
	Phatr3_J8113	*PtAureochrome1a*	*PtAureo1a*	-1.01	-0.06
	Phatr3_J15977	*PtAureochrome1b*	*PtAureo1b*	-2.01	-0.29
	Phatr3_J51933	*PtAureochrome1c*	*PtAureo1c*	2.04	0.09
	Phatr3_J15468	*PtAureochrome2*	*PtAureo2*	-2.73	-0.04
Group 1	Phatr3_EG02613	*PtUDP-Glucose-pyrophosphorylase*	*PtUDP*	6.56	-0.22
	Phatr3_J36821	*PtGlycerol-3-phosphate dehydrogenase*	*PtGPDH*	5.78	-0.01
	Phatr3_J55070	*PtHSF2*	*PtHSF2*	-4.13	0.93
	Phatr3_J38559	*PtPhatr3_J38559*	*PtJ38559*	-4.52	0.36
Group 2	Phatr3_J27278	*PtLhcx1*	*PtLhcx1*	3.31	0.14
	Phatr3_J44962	*PtbHLH1a_PAS*	*PtRitmo1a*	4.66	0.76
	Phatr3_J44963	*PtbHLH1b_PAS*	*PtRitmo1b*	3.62	1.02
	Phatr3_J48882	*PtLhcf15*	*PtLhcf15*	1.08	0.1
Group 3	Phatr3_J43744	*PtbZIP10*	*PtbZIP10*	2.47	0.69
	Phatr3_J54435	*PtbHLH2_PAS*	*PtbHLH2_PAS*	2.75	0.58
	Phatr3_J14908	*PtSigma70.5*	*PtSigma70.5*	3.33	0.91
	Phatr3_J44099	*PtHSF3.2b*	*PtHSF3.2b*	2.21	1.16
	Phatr3_J45206	*PtHSF3.2e*	*PtHSF3.2e*	1.73	0.29
